# A Phase III randomized controlled trial of Stereotactic Body Radiotherapy in localized prostate cancer

**DOI:** 10.1056/nejmoa2403365

**Published:** 2024-10-16

**Authors:** Nicholas van As, Clare Griffin, Alison Tree, Jaymini Patel, Peter Ostler, Hans van der Voet, Andrew Loblaw, William Chu, Daniel Ford, Shaun Tolan, Suneil Jain, Philip Camilleri, Kiran Kancherla, John Frew, Andrew Chan, Olivia Naismith, John Armstrong, John Staffurth, Alexander Martin, Ian Dayes, Paula Wells, Derek Price, Emily Williamson, Julia Pugh, Georgina Manning, Stephanie Brown, Stephanie Burnett, Emma Hall

**Affiliations:** 1 The Royal Marsden Hospital, London, UK; 2 The Institute of Cancer Research, London, UK; 3 Mount Vernon Cancer Centre, Northwood, UK; 4 The James Cook University Hospital, Middlesbrough, UK; 5 Odette Cancer Centre, Sunnybrook Health Sciences Centre, Toronto, ON, Canada; 6 University Hospitals Birmingham, Birmingham, UK; 7 The Clatterbridge Cancer Centre, Birkenhead, UK; 8 Queen’s University Belfast, Belfast, UK; 9 Churchill Hospital, Oxford, UK; 10 University Hospitals of Leicester, Leicester, UK; 11 Freeman Hospital, Newcastle, UK; 12 University Hospitals Coventry & Warwickshire, Coventry, UK; 13 Cancer Trials Ireland, Dublin, Ireland; 14 St Luke’s Radiation Oncology Network, St Lukes Hospital, Dublin, Ireland; 15 Velindre Cancer Centre, Cardiff, UK (JS); 16 Cambridge University Hospitals NHS Foundation Trust, Cambridge, UK; 17 Department of Oncology, McMaster University, Hamilton, ON, Canada; 18 St. Bartholomew’s Hospital, London, UK; 19 Patient and Public Representative, UK

## Abstract

**Background:**

PACE-B aims to demonstrate non-inferiority of Stereotactic Body Radiotherapy (SBRT) compared to conventionally or moderately hypo-fractionated regimens for biochemical and/or clinical failure for localised prostate cancer.

**Methods:**

PACE-B is an international phase III open-label randomised controlled trial. Men with stage T1-T2 prostate cancer, Gleason ≤3+4, PSA ≤20 ng/mL were randomized (1:1) to SBRT (36.25 gray (Gy) in 5 fractions (f) over 1-2 weeks) or control radiotherapy (CRT) (78Gy/39f over 7.5 weeks, or 62Gy/20f over 4 weeks). Androgen deprivation therapy was not permitted. The primary endpoint was freedom from biochemical/clinical failure with a critical hazard ratio for non-inferiority of 1.45. Analysis was by intention to treat.

**Results:**

874 patients were randomised from 38 centers (CRT=441, SBRT=433) between August 2012 and January 2018. Median age was 69.8 years, median PSA 8.0 ng/mL, NCCN risk group was 9.3% low, 90.7% intermediate. After 74.0 months median follow-up, 5-year biochemical/clinical failure free-rate (95% CI) was CRT: 94.6% (91.9%, 96.4%) vs SBRT: 95.8% (93.3%, 97.4%). SBRT was non-inferior to CRT with unadjusted hazard ratio 0.73 (90% CI: 0.48, 1.12; p-value for non-inferiority 0.004). To 5 years, cumulative rate of late RTOG grade 2 or worse genitourinary toxicity was CRT: 18.3% (95%CI 14.8, 22.5%) vs. SBRT: 26.9% (95%CI 22.8, 31.5%) (p<0.001) and for gastrointestinal toxicity was CRT: 10.2% (95%CI 7.7, 13.5%) vs. SBRT: 10.7% (95%CI 8.1, 14.2%) (p=0.94).

**Conclusion:**

Five-fraction SBRT is non-inferior to CRT for biochemical/clinical failure and is an efficacious treatment option for patients with low/intermediate risk localized prostate cancer as defined in this trial eligibility. (Funded by Accuray Incorporated and others; **ClinicalTrials.gov registration:** NCT01584258.)

## Introduction

Prostate cancer is a significant global healthcare challenge with nearly 1.5 million men diagnosed annually(1). In England in 2021, 12% of newly diagnosed prostate cancers were low risk and 29% intermediate risk(2). These men have a number of treatment options including radiotherapy, which is considered curative in the majority.

Innovations in image guidance and radiotherapy treatment delivery have enabled delivery of higher biologic doses of radiation, significantly improving oncologic outcomes and side effects associated with treatment(3–5). Hypofractionation, involving higher doses per treatment, is appealing due to its potential to maintain the efficacy of the treatment but reduce the total number of treatment sessions, which could make the treatment more attractive to patients and healthcare systems. Previous studies have confirmed non-inferiority for moderately hypofractionated radiotherapy compared with conventionally fractionated radiotherapy, and moderate hypofractionation has been established as a standard-of-care option (6–8). Stereotactic body radiotherapy (SBRT) builds on these developments to allow ultra-hypofractionated radiotherapy to be delivered with precision.

PACE (Prostate Advances in Comparative Evidence) is a platform trial evaluating five-fraction SBRT comprising three independently randomised cohorts of men with localised prostate cancer. PACE-A compares SBRT to surgery. PACE-B and PACE-C recruited men suitable for radical radiotherapy but not suitable for or unwilling to have radical prostatectomy. PACE-B included men with low and intermediate risk disease, not requiring hormone therapy, and has already demonstrated the safety of 5 fraction SBRT(9, 10). PACE-C included men with higher risk disease receiving androgen deprivation therapy. Here we report the primary outcome of PACE-B assessing non-inferiority of five-fraction SBRT compared to conventionally or moderately hypofractionated radiotherapy for biochemical or clinical failure.

## Methods

Eligible patients were ≥18 years, had histologically confirmed prostate adenocarcinoma, WHO performance status 0-2 and life expectancy >5 years. All participants had (clinical and/or MRI defined) T1 or T2 disease categorised based on NCCN criteria as low (Gleason 3+3 and PSA≤10ng/ml) or intermediate (at least one of the following factors: Gleason 3+4, PSA 10.1-20.0ng/ml) risk. Exclusion criteria included, primary Gleason grade 4 or higher disease, any NCCN high risk factors, previous pelvic radiotherapy, previous treatment for prostate cancer or bilateral hip prostheses.

NvA is the Chief Investigator and wrote the first draft. NvA, CG, JPa, GM, EH led manuscript writing; all other authors contributed to and reviewed the manuscript. No one who is not an author contributed to writing the manuscript. The authors accept responsibility for the data, its analysis, and adherence to the protocol.

### Trial design and randomisation

PACE-B was an international, phase III, open-label, non-inferiority randomized controlled trial. Participants were randomly assigned (1:1) to SBRT or control radiotherapy (CRT; conventionally or moderately hypofractionated radiotherapy). Randomization was performed centrally by the Institute of Cancer Research Clinical Trials and Statistics Unit (ICR-CTSU) using computer generated random permuted blocks (size 4 and 6), stratified by NCCN risk group (low vs intermediate) and randomizing center. Treatment was not masked.

### Treatment and assessments

For SBRT, insertion of three or more prostatic fiducial markers was recommended. Moderate bladder filling and bowel preparation (enemas) was advised for treatment planning. CT scan was completed with radiotherapy planning MRI recommended. CT and MRI scans were fused by fiducial matching. Clinical target volume was defined as prostate only for low-risk participants or prostate plus proximal 1cm of seminal vesicles for intermediate-risk participants. Clinical target volume to planning target volume margin was 4-5mm isotropic, except 3-5mm posteriorly. 36.25Gy in five fractions over 1-2 weeks (daily or alternate days) was delivered to 95% of the planning target volume and a secondary target dose of 40Gy to 95% of the clinical target volume only was delivered. SBRT was permitted on non-coplanar robotic linear accelerators and (since protocol v5.0, August 2014) conventional linear accelerator platforms. Further details are provided in The Radiotherapy Planning and Delivery Guidance in appendix S14. For CRT, initially the protocol mandated 78 gray (Gy) in 39 fractions (f) but following a protocol amendment (version 7.1 24/03/2016), 62Gy/20f was also permitted. Centers were required to choose a schedule to be used for all their participants. Androgen deprivation therapy (ADT) was not permitted.

PSA was recorded at 12 weeks, 6, 9, 12 months following treatment and annually thereafter. Participants were assessed using the Common Terminology Criteria for Adverse Events (CTCAE version 4.03.(5) and the Radiation Therapy Oncology Group (RTOG) assessment tool before treatment, every three months to 24 months, every 6 months years 2-5 and then annually to a maximum of 10 years.

Patient-reported outcomes (PRO) were assessed at baseline, months 6, 9, 12 and then annually to year 5 using the Expanded Prostate Cancer Index Composite short form (EPIC-26)(11), the International Prostate Symptom Score (IPSS), the Vaizey fecal incontinence score and the International Index of Erectile Function 5-Questionaire (IIEF-5; omitted at month 9). PROs were collected via paper questionnaires distributed in clinic or posted by centers. The protocol is available online (9) and at NEJM.org and a history of substantial amendments is in appendix S13.

### Trial oversight

PACE is an investigator-initiated trial approved by the London Chelsea Research Ethics Committee (11/LO/1915) in the UK and the relevant institutional review boards in Ireland and Canada. From protocol v5.0, August 2014, the trial was sponsored by the Royal Marsden NHS Foundation Trust and co-ordinated by the ICR-CTSU. Before this the trial was sponsored by Accuray. Accuray had no role in data collection (managed by a third party before February 2014) or statistical analysis (ICR-CTSU). The trial was conducted in accordance with the principles of Good Clinical Practice. Participants were recruited by their clinical teams and provided written, informed consent before enrolment. The Trial Management Group was overseen by an Independent Data Monitoring Committee and an independent Trial Steering Committee.

### Outcome measures

The primary outcome was biochemical or clinical failure. Biochemical failure was based on PSA rises (Phoenix criteria, with three consecutive rises required for failure before 24 months to rule out post-radiotherapy PSA “bounce”), commencement of ADT or date of orchidectomy and clinical failure was based on local recurrence, nodal recurrence, distant metastases and/or death from prostate cancer. The apriori defined time point of primary interest was 5 years. Participants without an event were censored on date of last PSA assessment. Secondary outcome measures included commencement of ADT, diagnosis of metastatic disease, disease-free survival, overall survival, clinician and patient assessed side effects.

### Statistical analysis

PACE-B was designed to assess non-inferiority of SBRT compared to CRT for biochemical failure. The sample size assumed 85% biochemical failure-free at 5 years with CRT. A non-inferiority margin of 6% at 5 years (critical hazard ratio (HR) 1.45; selected based on expert clinical opinion), 80% power, 5% one-sided significance and a 10% loss to follow-up allowance gave a sample size of 858 patients. Following recommendation by the Independent Data Monitoring Committee, the Trial Management Group and the Trial Steering Committee independently agreed (before any data release) to fix the critical HR at 1.45, if the observed control group biochemical failure-free estimate differed from that assumed. The protocol specified the principal analysis would take place once the required number of events had been observed (194) or a minimum of five years follow-up on all participants, whichever occurred first.

Efficacy analyses were on the intention to treat population with a primary endpoint sensitivity analysis conducted in the per protocol population. Kaplan Meier methods were used to estimate event rates. Estimates of treatment effect were made using unadjusted and adjusted (NCCN risk group) Cox regression models. For the primary outcome the HR is reported with the 90% confidence interval, in accordance with the 1-sided non-inferiority design. An HR< 1 favors SBRT. The absolute treatment difference in biochemical failure-free rates at 5 years are presented by applying the HR to the control group biochemical failure-free estimate and 90% confidence interval(12). The log-rank test was used to compare groups. HR with 95% confidence intervals is presented for all other efficacy outcomes (widths of confidence intervals have not been adjusted for multiplicity and should not be used in place of hypothesis testing). The proportional hazards assumption was assessed using Schoenfeld residuals and held for all time to event endpoints. A competing risks analysis was done for the primary outcome with non-prostate cancer deaths the competing event and differences between SBRT and CRT assessed using the Gray’s test. Pre-planned subgroup analyses of the primary outcome by NCCN risk group, age and Gleason score were conducted.

For clinician assessed toxicity (genitourinary (GU), gastrointestinal (GI) and erectile dysfunction), the proportion of grade ≥2 toxicity at 5 years is compared with Chi-squared or Fisher’s Exact tests. Cumulative incidence of late toxicity (defined as adverse events from 6 months post treatment) was estimated and time to first late adverse event was compared using Kaplan Meier methods. For PRO, EPIC-26 was analysed as composite scores (bowel, urinary, sexual and hormonal) and single item EPIC questions for overall bowel, urinary and sexual bother were presented at each time point assessed.

Analyses are based on a data snapshot taken on 11th September 2023 and were conducted using Stata version 17.0.

## Results

Between August 2012 and January 2018, 874 men (441 CRT, 433 SBRT) were randomized from 38 centers across the UK, Ireland and Canada (Appendix S2). 424/441 randomized to CRT and 414/433 randomized to SBRT received their allocated treatment; 25 received neither study treatment (Appendix S3). Eleven (8 CRT, 3 SBRT) participants were deemed ineligible but included in analyses. Reasons for ineligibility were: less than ten core biopsies being taken (n=5); prostate volume not measured within 6 months of randomization (n=3); significant urinary symptoms not identified until planning scan (n=1); no MRI done (n=1), biopsy not performed within 18 months of consent (n=1).

Baseline characteristics were well balanced between randomized groups (Table 1). Median age was 69.8 years (IQR 65.4, 74.0), median PSA ng/mL was 8.0 (IQR 5.9, 11.0) and 81/874 (9.3%) and 793/874 (90.7%) were low and intermediate NCCN risk groups respectively.

**Figure 1:**
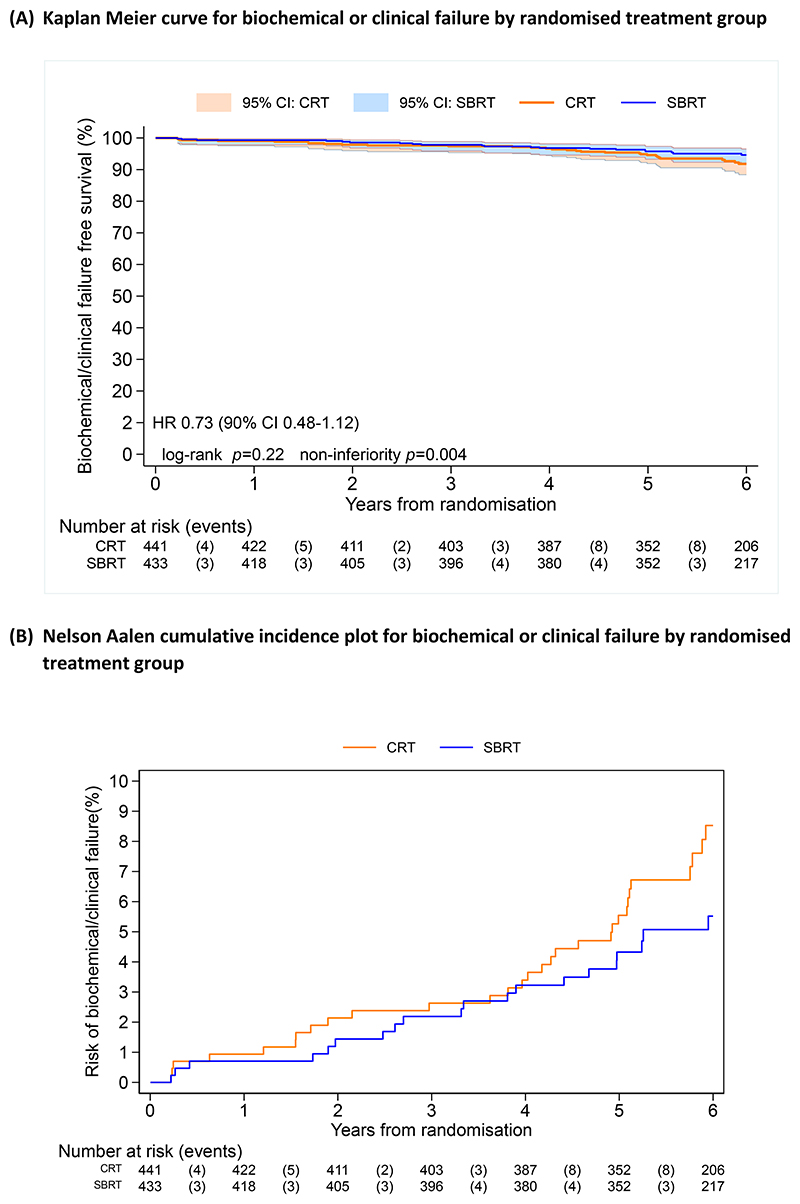

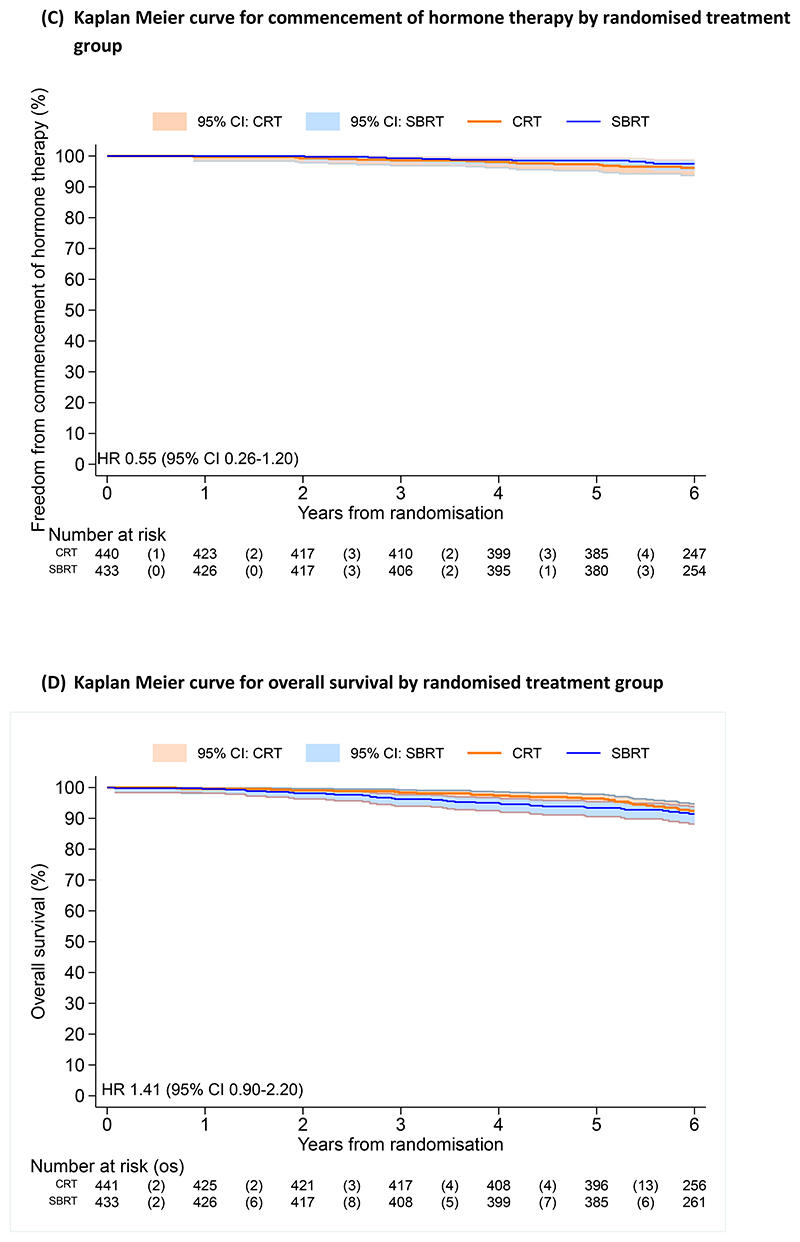
Efficacy outcomes (A) Kaplan Meier curve for biochemical or clinical failure by randomised treatment group, (B) Nelson Aalen cumulative incidence plot for biochemical or clinical failure by randomised treatment group, (C) Kaplan Meier curve for commencement of hormone therapy by treatment group, (D) Kaplan Meier curve for overall survival by randomised treatment group

SBRT was delivered over two weeks for 75% of patients on the CyberKnife for 41% of patients. Use of fiducials was more common with SBRT (73%) than CRT (57%) (Appendix S5). With a median follow-up of 74.0 months (IQR 64.8, 86.3), 36 and 26 biochemical failure events had occurred in the CRT and SBRT groups respectively. Five-year biochemical failure event-free rates (95% CI) were 94.6% (91.9, 96.4) for CRT and 95.8% (93.3, 97.4) for SBRT. SBRT was non-inferior to CRT with an unadjusted HR 0.73 (90%CI 0.48, 1.12), p-value for non-inferiority=0.004 (Figure 1A and Appendix S6). A test for superiority was not significant (HR 0.73; 95% CI: 0.44 1.21; p=0.22). The estimated absolute difference in the proportion of participants event free in the SBRT group compared with that in the CRT group at 5 years was: 1.43% (90% CI: -0.60, 2.78). An adjusted Cox model (HR 0.72; 90%CI 0.47, 1.10) and analysis in the per-protocol population (HR 0.65; 90%CI 0.42, 1.01) supported non-inferiority. Competing risks analysis indicated no evidence of a difference in biochemical failure rates between treatment groups (p=0.18). Pre-specified sub-group analysis showed no significant interactions with treatment group (Appendix S7).

Twenty-nine participants commenced hormone therapy (19 CRT, 10 SBRT), HR 0.55 (95%CI 0.26, 1.20), (Figure 1C and Appendix S4). Seventy-nine participants had died (33 CRT and 46 SBRT) with four deaths due to prostate cancer (two in each arm) and 28 to other primary cancers (Appendix S8); HR 1.41 (95%CI 0.90, 2.20), (Figure 1D and Appendix S6).

**Figure 2:**
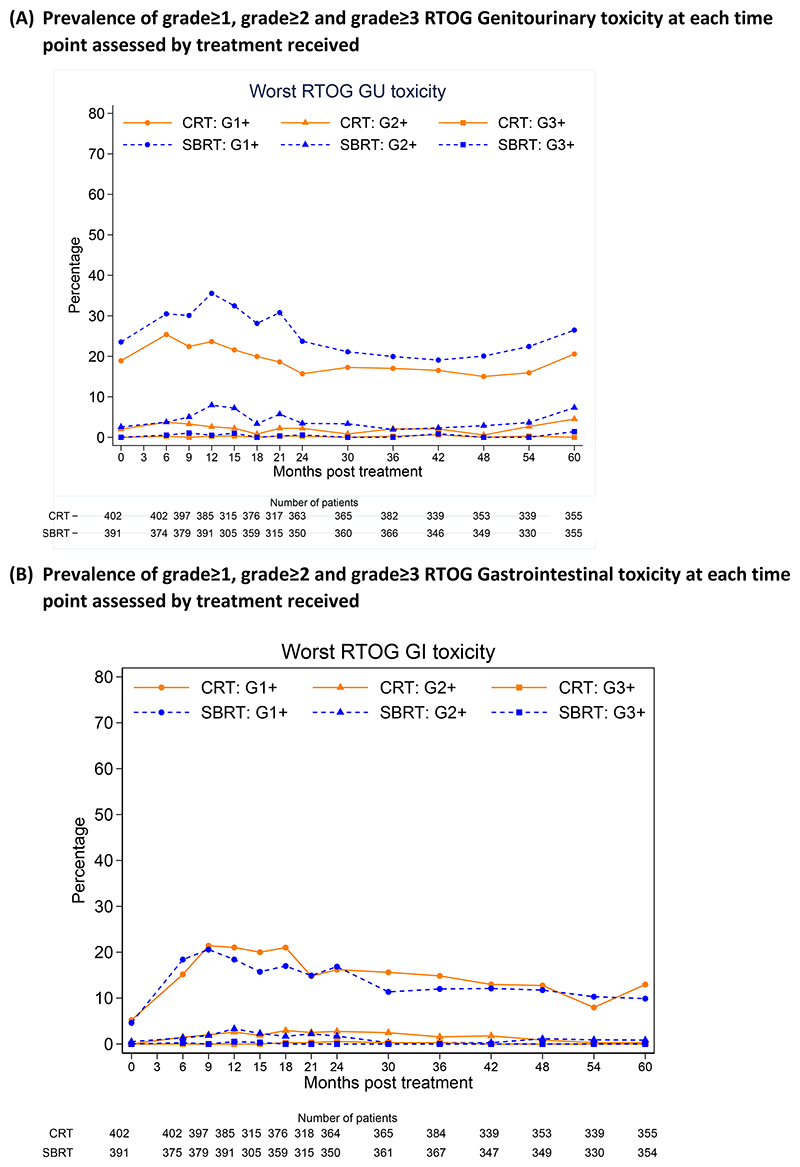

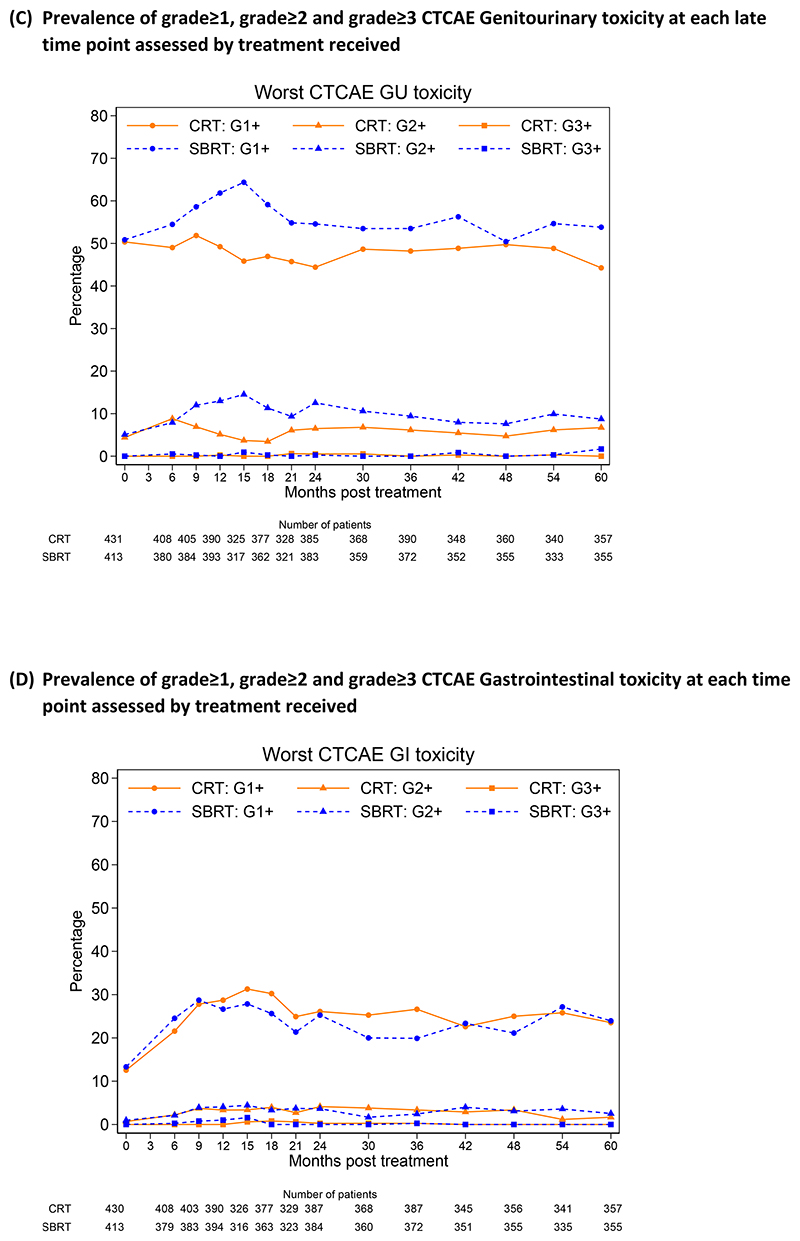

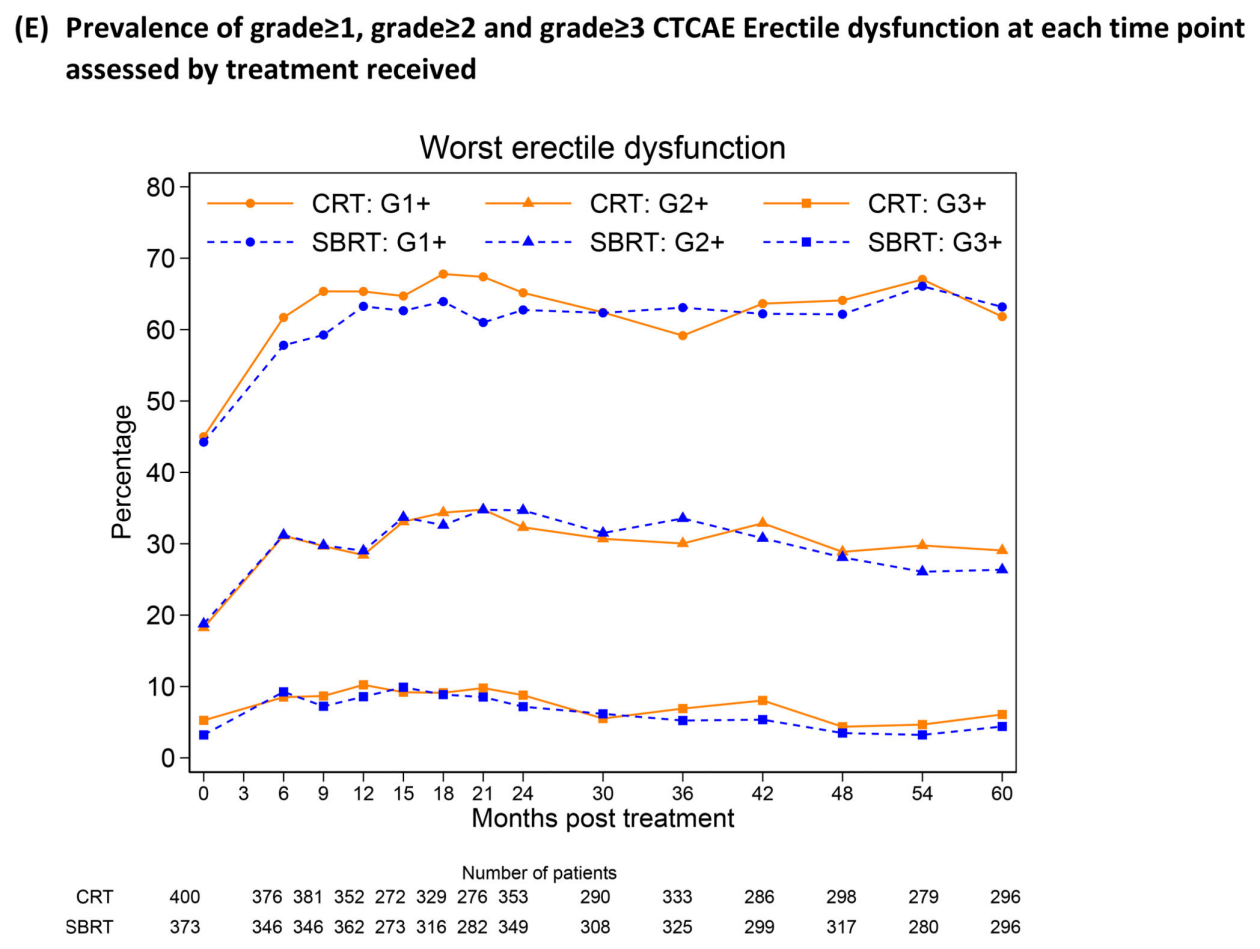
Prevalence of clinician reported RTOG and CTCAE assessed genitourinary, gastrointestinal toxicity and erectile dysfunction at each time point assessed by treatment received (A) Prevalence of grade≥1, grade≥2 and grade≥3 RTOG Genitourinary toxicity at each time point assessed by treatment received, (B) Prevalence of grade≥1, grade≥2 and grade≥3 RTOG Gastrointestinal toxicity at each time point assessed by treatment received (C) Prevalence of grade≥1, grade≥2 and grade≥3 CTCAE Genitourinary toxicity at each late time point assessed by treatment received (D) Prevalence of grade≥1, grade≥2 and grade≥3 CTCAE Gastrointestinal toxicity at each time point assessed by treatment received, (E) Prevalence of grade≥1, grade≥2 and grade≥3 CTCAE Erectile dysfunction at each time point assessed by treatment received

At 5 years, RTOG grade ≥2 GU toxicity was seen in 16/355(4.5%) participants who received CRT and 26/355 (7.3%) who received SBRT (p=0.11). CTCAE grade ≥2 GU toxicity was reported in 24/357 (6.7%) and 31/355 (8.7%) in the CRT and SBRT groups respectively at 5 years (p=0.32) (Figure 2, Appendix S9 & S11). Cumulative incidence rates differed for both RTOG and CTCAE grade≥2 GU toxicity (Appendix S10). For RTOG GU, incidence of late grade ≥2 events to 5 years was 18.3% (95%CI 14.8, 22.5%) and 26.9% (95%CI 22.8, 31.5%) for CRT and SBRT, respectively (HR 1.59 (95%CI 1.18, 2.12), p<0.001).

At 5 years, RTOG grade ≥2 GI toxicity was seen in 1/355(0.3%) receiving CRT and 3/354(0.8%) receiving SBRT (p=0.37) (Figure 2, Appendix S9 & S11). No difference in CTCAE GI grade ≥2 events was noted at 5 years: 6/357 (1.7%) CRT vs 9/355 (2.5%) SBRT (p=0.43) nor in cumulative incidence rates for RTOG or CTCAE grade ≥2 GI toxicity (Appendix S10). For RTOG GI, incidence rates of late grade ≥2 to 5 years were 10.2% (95%CI 7.7, 13.5%) and 10.7% (95%CI 8.1, 14.2%) for CRT and SBRT, respectively (HR 1.03 (95%CI 0.68, 1.56), p=0.94.

At 5 years, 86/296 (29.1%) CRT and 78/296 (26.4%) SBRT participants reported grade ≥2 CTCAE erectile dysfunction (p=0.46). Clinician reported grade ≥2 erectile symptoms were similar between treatment groups at baseline and were stable from 2 to 5 years after treatment (Figure 2, Appendix S9). Treatment related serious adverse events were reported in 12 participants (6 CRT; 6 SBRT).

**Figure 3:**
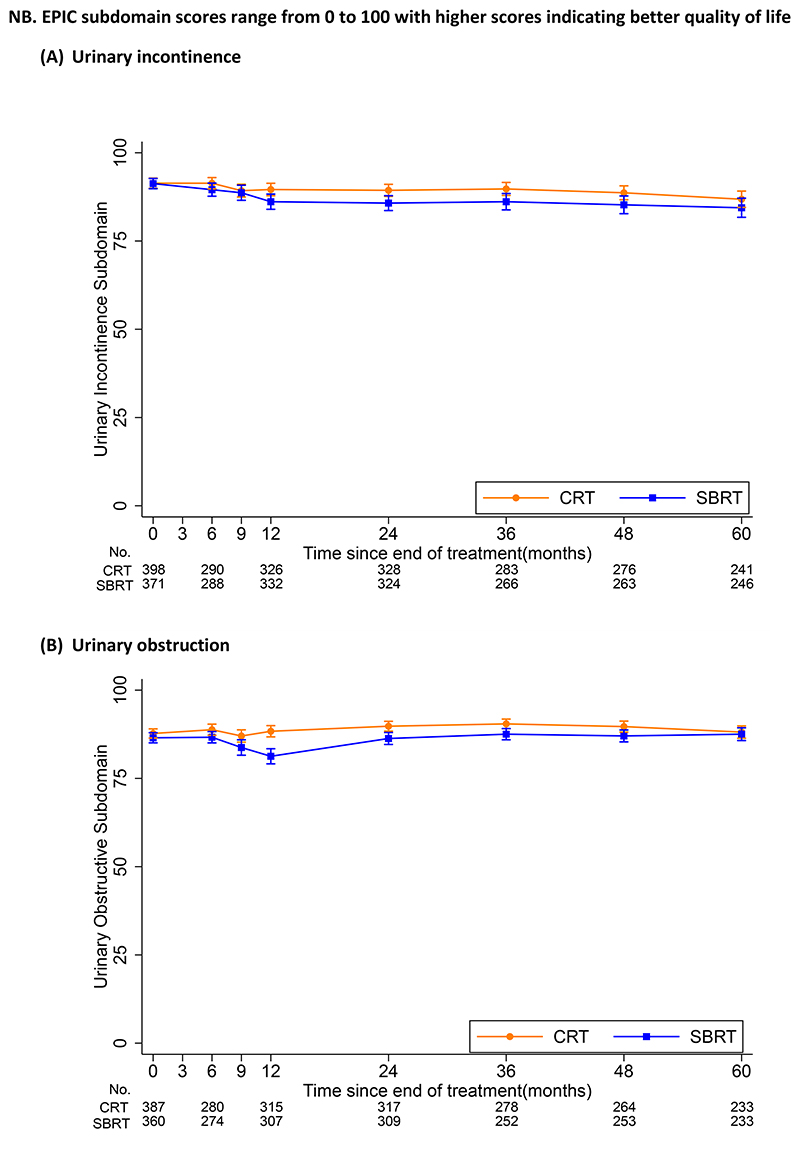

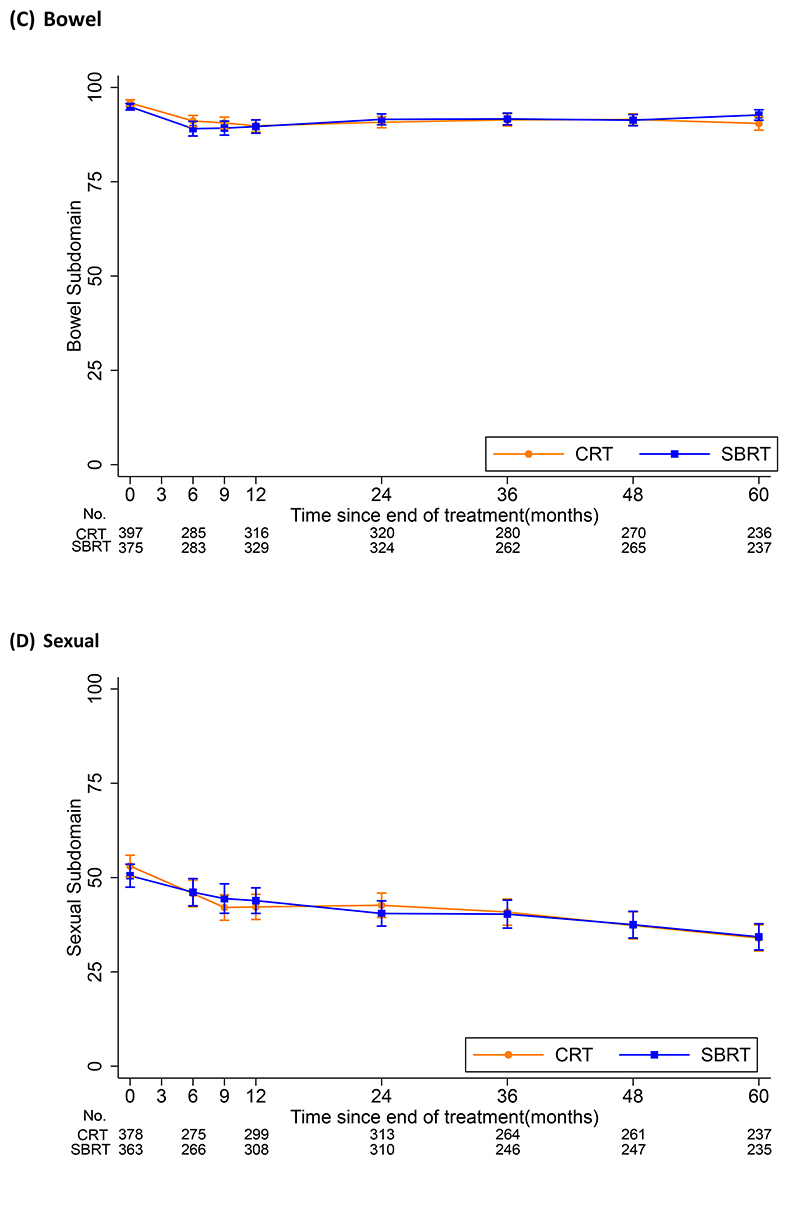

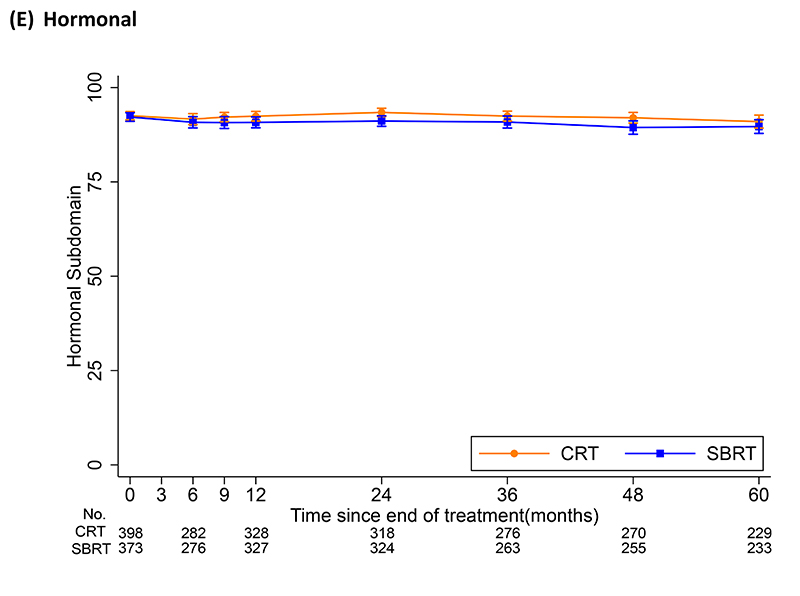
Patient reported mean EPIC subdomain composite scores by treatment received at each time point assessed Footnote-Error bars show 95% confidence interval for estimates of mean subdomain scores. Week 0 is the baseline toxicity score taken before start of radiotherapy. Abbreviations: EPIC-26 = Expanded Prostate Cancer Index Composite (26 question); CRT = Conventional radio therapy; SBRT = Stereotactic Body Radiotherapy

Participants reported stable urinary and bowel symptoms from 2 to 5 years, with little difference between treatment groups (Figure 3 and Appendix S12). At 5 years, median EPIC urinary incontinence scores of 100 (IQR 79.3, 100) and 96.9 (IQR 73.0, 100) were reported for CRT and SBRT respectively (p=0.45) (Appendix S12). No difference in EPIC urinary obstruction scores was noted at 5 years with median 93.8 (IQR 81.3, 100) for CRT and 93.8 (IQR 81.3, 100) for SBRT. Similar EPIC bowel subdomain scores were reported at 5 years with a median of 95.8 (IQR 87.5, 100) for CRT and 100 (IQR 87.5, 100) for SBRT (p=0.10). EPIC sexual subdomain scores declined from 2 to 5 years with no evidence of a difference between treatments at 5 years (p=0.87).

## Discussion

PACE-B has demonstrated non-inferiority of 5 fraction SBRT compared to moderately fractionated image-guided radiotherapy with excellent 5-year biochemical failure rates in both arms of the trial. The previous UK fractionation trial, CHHiP, included a slightly higher risk group and reported 5-year biochemical failure rate of 90.6% with moderately fractionated 60Gy/20f(6). The PACE-B biochemical failure-free rates of 95% and 96% for CRT and SBRT, respectively, were achieved without ADT and exceeded the expectations of the trial design.

These outcomes may reflect advancements in radiotherapy delivery, including improved image guidance with fiducial markers and cone beam CT, and enhanced treatment delivery with volumetric arc therapy, leading to greater accuracy and improved dose distributions. The trial protocol included comprehensive guidelines on treatment, clinical target volume and planning target volume delineation, planning, margins, image guidance, and treatment delivery. Adoption of five-fraction SBRT should follow these guidelines with appropriate quality assurance.

PACE-B is a randomized controlled trial that demonstrates the non-inferiority of SBRT in this context. Its results align with those of the HYPO-RT-PC phase 3 non-inferiority trial, which randomized 1,200 men between 78 Gy/39 fractions and 42.7 Gy/7 fractions over 2.5 weeks. The HYPO-RT-PC trial reported a 5-year failure-free survival rate of 84% in both arms (95% CI: 80–87) with an adjusted hazard ratio (HR) of 1.00 (95% CI: 0.76, 1.33; log-rank p=0.99). The differing outcomes between the two trials may be attributed to the inclusion of non-prostate cancer deaths as events in the HYPO-RT-PC study.

The strengths of the PACE-B trial include its large sample size and multicenter recruitment across three countries, with quality-assured radiotherapy delivered in both the control and experimental arms to a well-defined and homogeneous trial population. The absence of hormonal therapy in both arms ensures that the outcomes are not confounded by variable hormone therapy usage. At the time of trial design, the current NCCN classifications for favorable and unfavorable intermediate-risk disease did not exist. However, the majority of patients in the trial would now be classified as having unfavorable intermediate-risk cancer (Table 1). A limitation is that recommendations for five-fraction SBRT are restricted to men with similar risk features as those in the trial (Table 1). It remains unclear what proportion of the patients on this study would now optimally be managed with active surveillance given the absence of data that treatment influences overall survival in some patients with localized disease(13). Overtreatment is best avoided. The efficacy results of the PACE-C trial, which is evaluating the non-inferiority of five-fraction SBRT compared to 60 Gy/20 fractions in men with higher-risk disease requiring ADT, are awaited.

We have previously shown a significant increase in grade 2 or higher genitourinary (GU) toxicity at 2 years post-treatment (12% vs. 7%). The updated 5-year toxicity analysis indicates improvement in these symptoms, with no significant differences between the two arms at 5 years, and low overall levels of side effects. Patients should be informed of the higher medium-term risk of GU toxicity, especially those with significant lower urinary tract symptoms at baseline, who may achieve better symptomatic outcomes with 20-fraction IMRT. Patients with baseline lower urinary tract symptoms and/or significant acute toxicity are more likely to experience long-term toxicity, allowing for better patient selection for SBRT and careful counseling and monitoring of those with acute toxicity symptoms (14).

Prostate cancer radiotherapy represents a significant portion of the workload in radiotherapy departments globally. In England, over 16,000 patients received prostate radiotherapy in 2022, with an estimated 4,800 meeting PACE-B eligibility criteria(2). Transitioning these patients to a five-fraction regimen could reduce approximately 72,000 fractions across the UK. This regimen also minimizes the socioeconomic and psychological burdens of treatment. Additionally, NCCN-defined low-risk and some favorable intermediate-risk patients included in the trial could be considered for active surveillance.

The findings from PACE-B demonstrate that five-fraction SBRT is a robust and viable alternative to moderately fractionated radiotherapy for prostate cancer, demonstrating equivalent efficacy with enhanced convenience for patients. The high 5-year biochemical control rates, tolerability of treatment, coupled with the significant advancements in radiotherapy delivery, underscore the potential of SBRT’s use in prostate cancer treatment. The reduction in treatment fractions will alleviate burden on healthcare systems in addition to demonstrating excellent cancer control outcomes without the addition of hormonal treatment.

## Supplementary Material

Supplement

## Figures and Tables

**Table 1:** Baseline demographics by randomised treatment

Baseline Characteristics	CRT (N=441)		SBRT (N=433)		Total (N=874)	
	n	%	n	%	n	%
**Age at randomisation (years)**						
Median (IQR)	69.7	(65.5, 73.9)	69.8	(65.4, 74.1)	69.8	(65.4, 74.0)
(Range)		(48.1, 86.7)		(45.8, 84.5)		(45.8, 86.7)
**Ethnic origin**						
Black	26	(5.9)	35	(8.1)	61	(7.0)
East Asian	3	(0.7)	4	(0.9)	7	(0.8)
Mixed heritage	2	(0.5)	2	(0.5)	4	(0.5)
Southern Asian	10	(2.3)	20	(4.6)	30	(3.4)
White	393	(89.1)	367	(84.8)	760	(87.0)
Other*	7	(1.6)	5	(1.2)	12	(1.4)
**Family history of prostate cancer**						
No	326	(73.9)	312	(72.1)	638	(73.0)
Yes	88	(20.0)	89	(20.6)	177	(20.3)
Unknown	27	(6.1)	32	(7.4)	59	(6.8)
**WHO status**						
WHO status 0	391	(88.7)	389	(89.9)	780	(89.2)
WHO status 1	48	(10.9)	44	(10.2)	92	(10.5)
WHO status 2	2	(0.5)	0	(0.0)	2	(0.2)
**T-Stage** **						
T1c	81	(18.4)	82	(18.9)	163	(18.6)
T2a	133	(30.2)	105	(24.2)	238	(27.2)
T2b	59	(13.4)	81	(18.7)	140	(16.0)
T2c	168	(38.1)	162	(36.4)	330	(37.8)
Unknown	0		3	(0.8)	3	(0.4)
**Method of staging**						
Number with at least one staging method	441	(100)	430	(99.3)	871	(99.7)
DRE performed	166	(37.6)	156	(36.0)	322	(36.8)
TRUS performed	264	(59.9)	280	(64.7)	544	(62.2)
MRI of pelvis performed	359	(81.4)	339	(78.3)	698	(79.9)
**Gleason score**						
3+3	90	(20.4)	63	(14.5)	153	(17.5)
3+4	351	(79.6)	370	(85.3)	721	(82.5)
**PSA (ng/mL)**						
Median (IQR)	8.1	(6.3, 11.0)	7.9	(5.5, 10.9)	8.0	(5.9, 11.0)
N (Range)	441	(0.8, 20.0)	433	(0.5, 20.0)	874	(0.5, 20.0)
PSA<10	303	(68.7)	297	(68.6)	600	(68.7)
PSA 10-20	138	(31.3)	136	(31.4)	274	(31.6)
**Percentage positive cores**						
<50%	304	(68.9)	287	(66.3)	591	(67.6)
≥50%	137	(31.1)	146	(33.7)	283	(32.4)
**NCCN risk group**						
Low	41	(9.3)	32	(7.4)	73	(8.4)
Intermediate	400	(90.7)	401	(92.6)	801	(91.7)
Favorable	106	(26.5)	86	(21.5)	192	(24.0)
Unfavorable	294	(73.5)	315	(78.6)	609	(76.0)
**Prostate volume**						
<40 mL	163	(37.0)	192	(44.3)	355	(40.6)
40 - <80 mL	223	(50.6)	198	(45.7)	421	(48.2)
80+ mL	28	(6.3)	22	(5.3)	51	(5.8)
Unknown	27	(6.1)	20	(4.6)	47	(5.4)
**Testosterone [μmol/L]**						
Median (IQR)	11.3	(8.7, 15.0)	11.5	(9.0, 15.0)	11.3	(8.9, 15.0)
N (Range)	407	(0.4, 30.6)	403	(0.4, 30.5)	810	(0.4, 30.6)
Unknown	34		30		64	
**Baseline IPSS grade**						
None	21	(4.8)	16	(3.7)	37	(4.3)
Mild	197	(44.7)	202	(46.7)	399	(45.7)
Moderate	141	(32.0)	136	(31.4)	277	(31.7)
Severe	23	(5.2)	20	(4.6)	43	(4.9)
Unknown	59	(13.4)	59	(13.6)	118	(13.5)
**Time from diagnosis to randomisation (weeks)** ***						
Median (IQR)	11.0	(6.9, 17.0)	9.9	(6.6, 16.1)	10.1	(6.7, 16.6)
(Range)		(0.9, 335.0)		(0.1, 225.0)		(0.1, 335.0)

Data presented as n (%) or median (IQR) (range). CRT=control radiotherapy, SBRT=stereotactic body radiotherapy, NCCN=National Comprehensive Cancer Network, PSA=prostate specific antigen, IPSS=International Prostate Symptom Score, TRUS=transrectal ultrasound, DRE=digital rectal exam.

* 6-not disclosed, 8 other ethnic group

** Where different staging techniques resulted in different T-stage, highest T-stage is used.

*** According to protocol, histological confirmation of prostate adenocarcinoma within the last 18 months unless on active surveillance and not clinically indicated

## References

[1] (2018). Prostate Cancer - Statistics.

[2] (2024). National Prostate Cancer Audit: State of the Nation Report.

[3] Michalski JM, Moughan J, Purdy JA, Bruner DW, Amin M, Bahary JP (2023). Long-Term Outcomes of NRG/RTOG 0126, a Randomized Trial of High Dose (79.2Gy) vs. Standard Dose (70.2Gy) Radiation Therapy (RT) for Men with Localized Prostate Cancer.

[4] Hennequin C, Sargos P, Roca L, Silva M, Latorzeff I, Peiffert D (2024). Long-term results of dose escalation (80 vs 70 Gy) combined with long-term androgen deprivation in high-risk prostate cancers: GETUG-AFU 18 randomized trial. Journal of Clinical Oncology.

[5] Michalski JM, Moughan J, Purdy JA, Bosch W, Bruner DW, Bahary JP (2018). Effect of Standard vs Dose-Escalated Radiation Therapy for Patients With Intermediate-Risk Prostate Cancer, The NRG Oncology RTOG 0126 Randomized Clinical Trial. JAMA Oncology.

[6] Dearnaley D, Syndikus I, Mossop H, Khoo V, Birtle A, Bloomfield D (2016). Conventional versus hypofractionated high-dose intensity-modulated radiotherapy for prostate cancer: 5-year outcomes of the randomised, non-inferiority, phase 3 CHHiP trial. The Lancet Oncology.

[7] Lee WR, Dignam J, Amin MB, Bruner DW, Low D, Swanson GP (2016). Randomized Phase III Noninferiority Study Comparing Two Radiotherapy Fractionation Schedules in Patients With Low-Risk Prostate Cancer. Journal of Clinical Oncology.

[8] Catton CN, Lukka H, Gu C, MArtin JM, Supiot S, Chung PWM (2017). Randomized Trial of a Hypofractionated Radiation Regimen for the Treatment of Localized Prostate Cancer. Journal of Clinical Oncology.

[9] Brand DH, Tree AC, Ostler P, Voet Hvd, Loblaw A, Chu W (2019). Intensity-modulated fractionated radiotherapy versus stereotactic body radiotherapy for prostate cancer (PACE-B): acute toxicity findings from an international, randomised, open-label, phase 3, non-inferiority trial. The Lancet Oncology.

[10] Tree AC, Ostler P, Voet Hvd, Chu W, Loblaw A, Ford D (2022). Intensity-modulated radiotherapy versus stereotactic body radiotherapy for prostate cancer (PACE-B): 2-year toxicity results from an open-label, randomised, phase 3, non-inferiority trial. The Lancet Oncology.

[11] Szymanski KM, Wei JT, Dunn RL, Sanda MG (2010). Development and Validation of an Abbreviated Version of the Expanded Prostate Cancer Index Composite Instrument for Measuring Health-related Quality of Life Among Prostate Cancer Survivors. Urology.

[12] Altman DG (1999). Calculating the number needed to treat for trials where the outcome is time to an event. The British Medical Journal.

[13] Hamdy FC, Donovan HL, Lane JA, Metcalfe C, Davis M, Turner EL (2023). Fifteen-year outcomes after monitoring, surgery, or radiotherapy for prostate cancer. N Engl J Med.

[14] Ratnakumaran R, Hinder V, Brand DH, Staffurth J, Hall E, As Nv (2023). The Association between Acute and Late Genitourinary and Gastrointestinal Toxicities: An Analysis of the PACE B Study. Cancers.

