# Supplementary material for: A Phase III randomized controlled trial of Stereotactic Body Radiotherapy in localized prostate cancer
Source: N Engl J Med. Author manuscript; Available in PMC 2024 Oct 17. (PMC7616714; doi:10.1056/nejmoa2403365)
Supplement: Supplement [file EMS197589-supplement-Supplement.pdf]

Supplementary Appendix

A Phase III randomized controlled trial of Stereotactic Body Radiotherapy in localized prostate cancer

Authorship

Nicholas van As, MD(Res)[1, 2], Clare Griffin, MSc[2], Alison Tree, MD(Res)[1, 2], Jaymini Patel, PhD[2], Peter Ostler, FRCR[3], Hans van der Voet, MD[4], Andrew Loblaw, MD[5], William Chu, MD[5], Daniel Ford, FRCR[6], Shaun Tolan, MB BCh[7], Suneil Jain, PhD[8], Philip Camilleri, FRCR[9], Kiran Kancherla, FRCR[10], John Frew, FRCR[11], Andrew Chan, FRCR[12], Olivia Naismith, MSc[1], John Armstrong, FFRRCSI[13, 14], John Staffurth, MD[15], Alexander Martin, MD(Res)[16], Ian Dayes, MD[17], Paula Wells, PhD[18], Derek Price[19], Emily Williamson, MSc[2] Julia Pugh, CIM Dip[2], Georgina Manning, BA Hons[2], Stephanie Brown, PhD[2], Stephanie Burnett, BSc[2], Emma Hall, PhD[2]

Affiliations:

1. The Royal Marsden Hospital, London, UK
2. The Institute of Cancer Research, London, UK
3. Mount Vernon Cancer Centre, Northwood, UK
4. The James Cook University Hospital, Middlesbrough, UK
5. Odette Cancer Centre, Sunnybrook Health Sciences Centre, Toronto, ON, Canada
6. University Hospitals Birmingham, Birmingham, UK
7. The Clatterbridge Cancer Centre, Birkenhead, UK
8. Queen's University Belfast, Belfast, UK
9. Churchill Hospital, Oxford, UK
10. University Hospitals of Leicester, Leicester, UK
11. Freeman Hospital, Newcastle, UK
12. University Hospitals Coventry & Warwickshire, Coventry, UK
13. Cancer Trials Ireland, Dublin, Ireland
14. St Luke’s Radiation Oncology Network, St Lukes Hospital, Dublin, Ireland
15. Velindre Cancer Centre, Cardiff, UK (JS).
16. Cambridge University Hospitals NHS Foundation Trust, Cambridge, UK

17. Department of Oncology, McMaster University, Hamilton, ON, Canada

18. St. Bartholomew's Hospital, London, UK

19. Patient and Public Representative, UK

Table of Contents

S1. PACE Trial oversight committees (past and current)........ 4
S2. PACE: Recruiting centres across the UK, Ireland and Canada 6
S3. PACE: B Consort flow chart........ 7
S4. Concomitant medications used at baseline by randomised treatment group........ 8
S5. PACE: Radiotherapy treatment delivery by treatment received........ 9
S6. Efficacy outcome measures by randomised treatment group 10
S7. Univariable subgroup analyses of biochemical and/or clinical failure comparing SBRT with CRT........ 11
S8. Tabulation of causes of death 12
S9. Clinician reported toxicity at 5 years post treatment by treatment received........ 13
S10. Cumulative incidence of Grade 2+ events by 5 years........ 14
S11. Stacked bar charts illustrating distribution of genitourinary and gastrointestinal toxicity assessed using RTOG and CTCAE........ 16
S12. EPIC subdomain scores at baseline and 5 years by treatment received 19
S13. Summary of PACE Protocol Substantial Amendments........ 22
S14. Radiotherapy Planning and Delivery Guidelines 25

S1. PACE Trial oversight committees (past and current)

Independent Data Monitoring Committee:

Chair: Professor Søren Bentzen (Director of the Division of Biostatistics & Bioinformatics), University of Maryland School of Medicine.

Professor Joe O'Sullivan (Professor of Clinical Oncology), Belfast City Hospital, UK.

Mr Raj Persad (Consultant Urological Surgeon), Bristol Royal Infirmary and Southmead Hospital, UK.

Independent Trial Steering Committee:

Chair: Professor Anthony Zietman: Boston, Massachusetts, USA.

Professor Alberto Bossi: Gustave Roussy Cancer Institute, Villejuif, France.

Professor Alberto Briganti: San Raffaele University Hospital, Milan, Italy.

Professor Ann Henry: St. James University Hospital, Leeds, UK.

Professor John Norrie: University of Aberdeen, UK.

Dr Piet Ost: Ghent University Hospital, Belgium.

Dr Gert DeMeerleer: Ghent University Hospital, Belgium.

Members added since November 2023:

Professor Søren Bentzen: University of Maryland School of Medicine, USA.

Professor Robert Bristow: The University of Manchester, UK.

Dr Vivian Cosgrove: Leeds Cancer Centre, UK.

Manoj Mistry: Patient and Public Involvement (PPI) representative.

Helen Bulbeck: PPI representative.

Bob White: PPI representative.

Trial Management Group:

Chair: Professor Nicolas van As: The Royal Marsden Hospital, London, UK; The Institute of Cancer Research, London, UK.

Dr Alison Tree: The Royal Marsden Hospital, London, UK; The Institute of Cancer Research, London, UK.

Professor Emma Hall: The Institute of Cancer Research, London, UK.

Professor John Armstrong: Cancer Trials Ireland, Dublin, Ireland.

Dr Doug Brand: The Royal Marsden Hospital, London, UK.

Dr William Chu: Sunnybrook, Canada.

Dr David Dodds: Beatson Cancer Centre, Glasgow, UK.

Aileen Duffton: Beatson Cancer Centre, Glasgow, UK.

Dr Daniel Ford: University Hospitals Birmingham, UK.

Dr Daniel Henderson: The Royal Marsden Hospital, London, UK.

Professor Suneil Jain: Belfast City Hospital, UK. a

Dr Andrew Loblaw: Sunnybrook, Canada.

Professor Eric Lartigau: Oscar Lambret Centre, France.

Dr Alexander Martin: Addenbrooke's Hospital, Cambridge, UK.

Dr Kirsty Morrison: The Royal Marsden Hospital, London, UK.

Dr Andrew Chan: University Hospitals Coventry & Warwickshire, Coventry, UK.

Olivia Naismith: The Royal Marsden Hospital, London, UK; Radiotherapy Trials QA Group, London, UK.

Dr Peter Ostler: Mount Vernon Cancer Centre, Northwood, UK.

Derek Price: PPI representative.

Sonia Patterson: PPI representative.

Professor John Staffurth: Velindre Cancer Centre, UK.

Dr Shaun Tolan: The Clatterbridge Cancer Centre, Liverpool, UK

Alan Thompson: The Royal Marsden Hospital, London, UK.

Dr Hans van der Voet: The James Cook University Hospital, Middlesbrough, UK.

Mathias Winkler: The Royal Marsden Hospital, London, UK.

Declan Cahill: The Royal Marsden Hospital, London, UK.

Dr Stephanie Brown: The Institute of Cancer Research, London, UK.

Stephanie Burnett: The Institute of Cancer Research, London, UK.

Georgina Manning: The Institute of Cancer Research, London, UK.

Clare Griffin: The Institute of Cancer Research, London, UK.

Jaymini Patel: The Institute of Cancer Research, London, UK.

Victoria Hinder: The Institute of Cancer Research, London, UK.

Clare Cruickshank: The Institute of Cancer Research, London, UK.

Muneeb Mahmood: The Institute of Cancer Research, London, UK.

Keely Tomkinson: The Institute of Cancer Research, London, UK.

Hassan Nawrozzadeh: The Institute of Cancer Research, London UK.

Helen Mossop: The Institute of Cancer Research, London UK.

S2. PACE: Recruiting centres across the UK, Ireland and Canada

Centre Name	PI	Date site open	Centre months open	Number patients recruited
Royal Marsden Hospital, London, UK	Dr Van As	Aug 12	65	**172**
Mount Vernon Hospital, Northwood, UK	Dr Ostler	Jan 13	60	**114**
James Cook University Hospital, Middlesbrough, UK	Dr Van der Voet	20 Oct 15	26	**110**
Odette Cancer Centre, Toronto, Canada	Dr Chu	10 Feb 16	22	**83**
Churchill Hospital, Oxford, UK	Dr Camilleri	12 Oct 15	26	**41**
Queen Elizabeth Hospital, Birmingham, UK	Dr Ford	13 Nov 15	25	**36**
Leicester Royal Infirmary, Leicester, UK	Dr Kancherla	25 Jul 16	17	**34**
Freeman Hospital, Newcastle upon Tyne, UK	Dr Frew	21 Oct 15	26	**30**
UH Coventry & Warwickshire, Coventry, UK	Dr Chan	03 Dec 15	25	**30**
Clatterbridge Cancer Centre, Wirral, UK	Dr Tolan	24 May 16	19	**25**
Juravinski Cancer Centre, Hamilton, Canada	Dr Dayes	27 Jan 17	11	**24**
Belfast City Hospital, Belfast, UK	Dr Jain	21 Oct 15	26	**21**
St Bartholomew's Hospital, London, UK	Dr Wells	23 Aug 16	16	**17**
Hôspital Charles-LeMoyne, Longueuil, Canada	Dr Lymberiou	23 May 17	7	**14**
Addenbrooke's Hospital, Cambridge, UK	Dr Martin	13 Jan 16	23	**13**
Nottingham City Hospital, Nottingham, UK	Dr Saunders	21 Jun 16	18	**11**
Royal Free Hospital, London, UK	Dr Vilarino-Varela	18 Jan 17	11	**11**
Hôspital Maisonneuve-Rosemont, Montreal, Canada	Dr Vavassis	14 Sep 17	3	**11**
Walker Family Cancer Centre, St. Catharines, Canada	Dr Tsakiridis	03 Apr 17	9	**9**
Hinchingbrooke Hospital, Huntingdon, UK	Dr Russell	20 Jan 16	23	**7**
Northeast Cancer Centre, Sudbury, Canada	Dr Carlson	19 Oct 16	14	**7**
London Health Sciences Centre, Ottawa, Canada	Dr Rodrigues	11 Aug 17	4	**7**
Velindre Cancer Centre, Wales, UK	Dr Tanguay	28 Jun 17	6	**6**
Sunderland Royal Hospital, Sunderland, UK	Dr Iqbal	03 Jun 16	19	**5**
Charing Cross Hospital, London, UK	Mr Winkler	13 Sep 16	15	**5**
Ottawa Hospital, Ottawa, Canada	Dr Morgan	23 Aug 17	4	**5**
Beacon Hospital, Dublin, Ireland	Dr Mihai	03 Mar 17	10	**4**
Lakeridge Health, Oshawa, Canada	Dr Li	09 Mar 17	9	**4**
Weston Park Hospital, Sheffield, UK	Dr Din	16 Aug 17	4	**4**
Lincoln County Hospital, Lincoln, UK	Dr Panades	14 Mar 17	9	**3**
Norfolk & Norwich UH, Norwich, UK	Dr Wade	01 Jun 17	7	**3**
West Suffolk Hospital, Bury Saint Edmunds, UK	Dr Martin	21 Jun 16	18	**2**
St Luke's Research Oncology Network, Dublin, Ireland	Dr Armstrong	18 Aug 17	4	**2**
Pilgrim Hospital, Boston, UK	Dr Panades	30 Mar 17	9	**1**
Glan Clwyd, North Wales, UK	Dr Oommen	07 Aug 17	4	**1**
University College London Hospital, London, UK	Dr Mitra	03 Mar 17	10	**0**
The Beatson, Glasgow, UK	Dr Dodds	04 Oct 17	3	**0**
**Total**			**615**	**874**

S3. PACE: B Consort flow chart

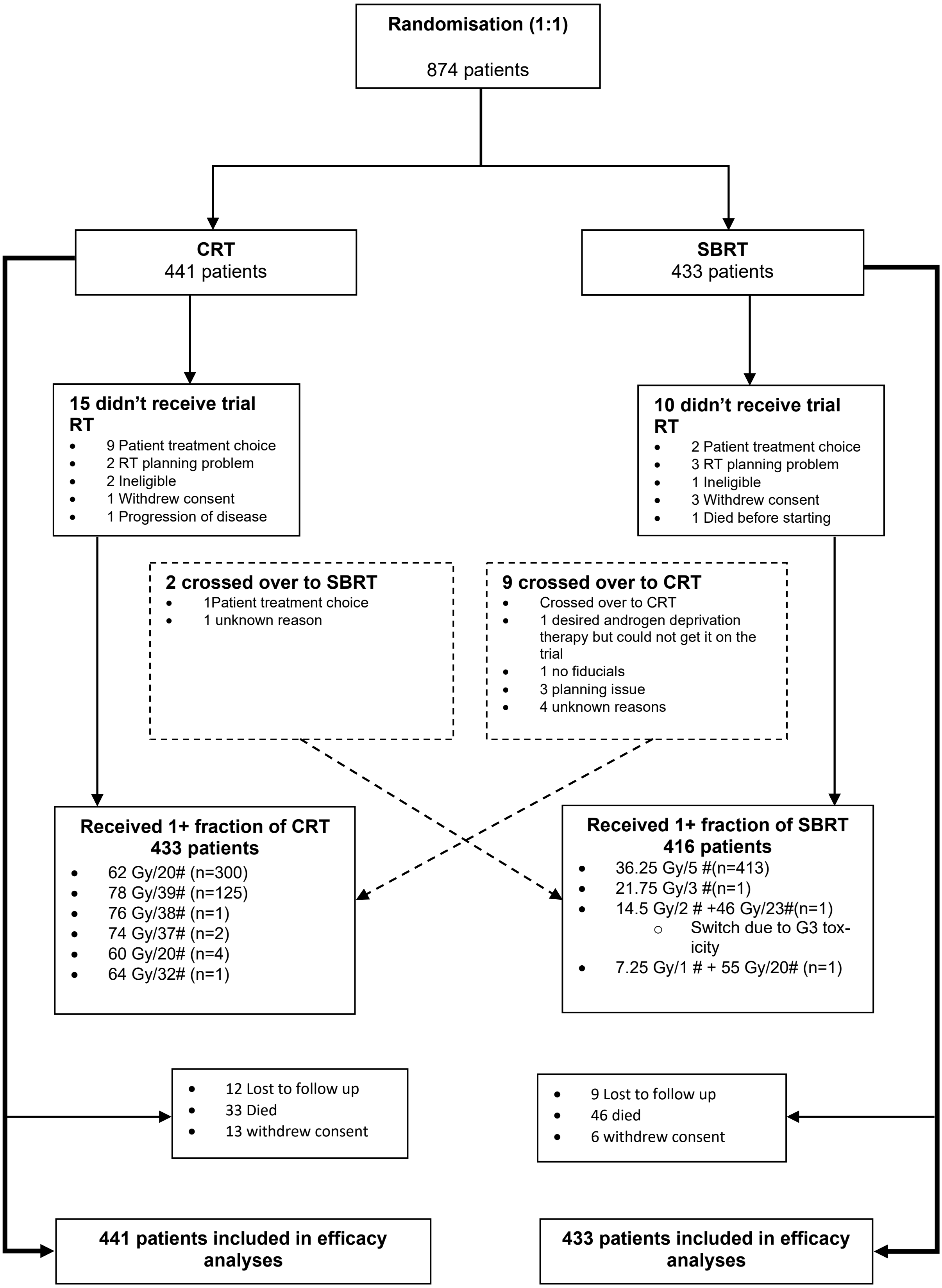

S4. Concomitant medications used at baseline by randomised treatment group

**Alpha blockers at randomisation**						
Yes	68	(15.4)	72	(16.6)	140	(16.0)
No	368	(83.4)	356	(82.2)	724	(82.8)
Unknown	5	(1.1)	5	(1.2)	10	(1.1)
**Aspirin at randomisation**						
Yes	80	(18.1)	73	(16.9)	153	(17.5)
No	356	(80.7)	354	(81.8)	710	(81.2)
Unknown	5	(1.1)	6	(1.4)	11	(1.3)
**Statin at randomisation**						
Yes	159	(36.1)	137	(31.6)	296	(33.9)
No	277	(62.8)	289	(66.7)	566	(64.8)
Unknown	4	(0.9)	7	(1.6)	12	(1.4)
**Anticholinergic for bladder symptoms at randomisation**						
Yes	14	(3.2)	12	(2.8)	26	(3.0)
No	423	(95.9)	415	(95.8)	838	(95.9)
Unknown	4	(0.9)	6	(1.4)	10	(1.1)
**5-alpha reductase inhibitors at randomisation**						
Yes	9	(2.0)	11	(2.5)	20	(2.3)
No	422	(95.7)	402	(92.8)	824	(94.3)
Unknown	10	(2.3)	20	(4.6)	30	(3.4)
**Phosphodiesterase-5 inhibitors for erectile dysfunction at randomisation**						
Yes	13	(3.0)	6	(1.4)	19	(2.3)
No	408	(94.9)	393	(94.9)	801	(94.9)
Unknown	9	(2.1)	15	(3.6)	24	(2.8)

S5. PACE: Radiotherapy treatment delivery by treatment received

Treatment Characteristics	CRT (N=433)		SBRT (N=415)		Total (N=848)	
	n	%	n	%	n	%
**Fiducial markers inserted?**						
Yes	245	57	303	73	548	65
No	188	43	112	27	300	35
**Planned Radiotherapy technique**						
Static field IMRT	92	21	1*	0	93	11
VMAT	322	74	242	59	564	67
Cyberknife	0	0	169	41	169	20
Other	19	4	3	1	22	2
**Overall treatment time**						
1 week	0	0	87	21	87	10
2 weeks	0	0	311	75	311	37
3 weeks	0	0	17	4	17	2
4 weeks	135	31	0	0	135	16
5 weeks	167	39	0	0	167	20
6 weeks	2	0	0	0	2	0
7 weeks	1	0	0	0	1	0
8 weeks	60	14	0	0	60	7
9 weeks	68	16	0	0	68	8

*Patient randomised to CRT but received SBRT

S6. Efficacy outcome measures by randomised treatment group

Endpoint	Number of events n/patients; %	Kaplan Meier 5-year estimate proportion event free 95% CI	Unadjusted HR 95%CI	Adjusted HR[1] 95% CI
**Biochemical or clinical failure**				
CRT	36/441 (8.2)	94.6 (91.9, 96.4)	0.73 (0.44, 1.21)	0.72 (0.43, 1.19)
SBRT	26/433 (6.0)	95.8 (93.3, 97.4)		
**Overall survival**				
CRT	33/441 (7.5)	96.4 (94.2,97.8)	1.41 (0.90, 2.20)	1.40 (0.89, 2.19)
SBRT	46/433 (10.6)	93.4 (90.6, 95.4)		
**Commencement of hormones**				
CRT	19/441 (4.3)	97.3 (95.3,98.5)	0.55 (0.26,1.20)	0.55 (0.25, 1.19)
SBRT	10/433 (2.3)	98.5 (96.8, 99.3)		
**Distant disease**				
CRT	7/441 (1.6)	98.8 (97.1,99.5)	0.58 (0.17,1.99)	0.57 (0.16, 1.96)
SBRT	4/433 (0.9)	99.3 (97.7, 99.8)		
**Disease free survival**				
CRT	62/441 (14.0)	91.5 (88.3,93.8)	1.10 (0.78, 1.55)	1.50 (0.74, 3.08)
SBRT	68/433 (15.7)	89.6 (86.3, 92.2)		
**Disease specific survival**				
CRT	2/441 (0.5)	100[2]	0.94 (0.13, 6.69)	0.90 (0.13, 6.42)
SBRT	2/433 (0.5)	100[2]		

[1] Cox model adjusted for NCCN risk group

[2] All prostate cancer deaths occurred after 5 years

Events contributing to the primary endpoint by randomised treatment group

Type of first event N (%)	**CRT N=441**	**SBRT N=433**	**Total N=874**
**Primary endpoint event**	**36 (8.2)**	**26 (6.0)**	**62 (7.1)**
Biochemical failure	27 (6.1)	20 (4.6)	47 (5.4)
Local recurrence	2 (0.5)	2 (0.5)	4 (0.5)
Nodal recurrence	4 (0.9)	1 (0.2)	5 (0.6)
Distant metastases	1 (0.2)	1 (0.2)	2 (0.2)
Prostate cancer death	2 (0.5)	2 (0.5)	4 (0.5)

S7. Univariable subgroup analyses of biochemical and/or clinical failure comparing SBRT with CRT

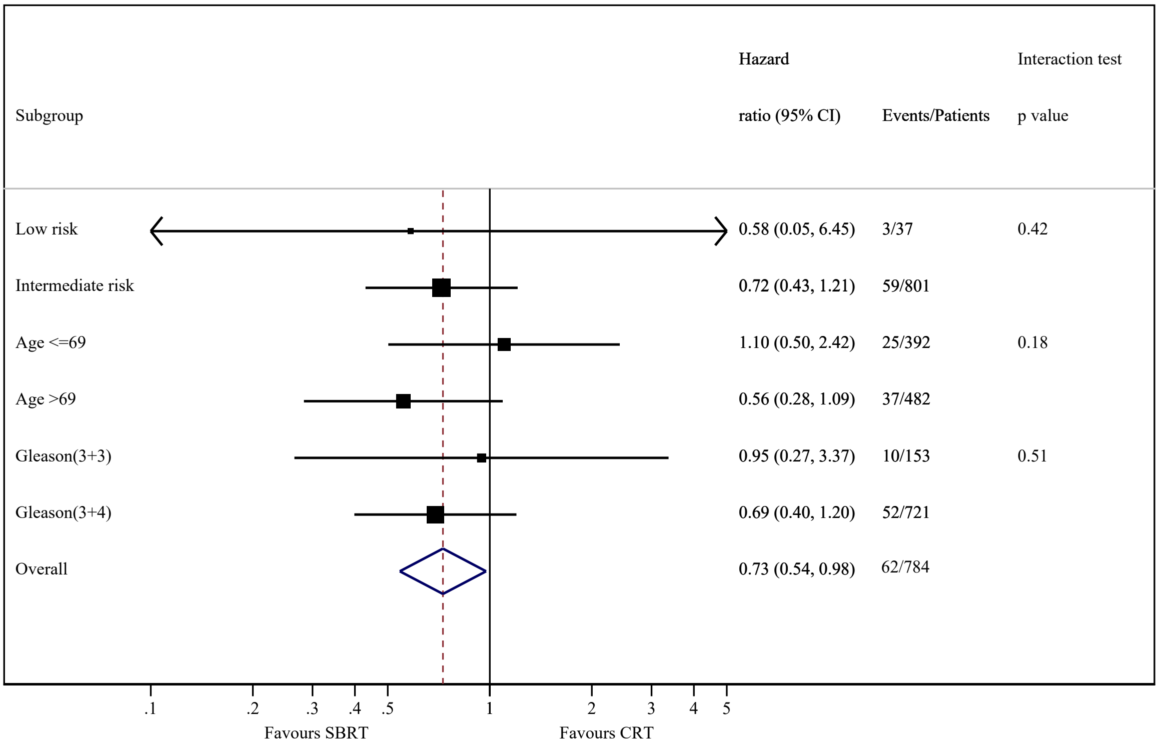

Hazard ratio and 95% confidence intervals are shown for the primary end point event of biochemical/clinical failure between two groups.

Note: There were very small number of biochemical and/or clinical failure events reported in the low risk group (CRT (2), SBRT (1))

S8. Tabulation of causes of death

	CRT (N=441)		SBRT (N=433)		Total (N=874)	
	**No.**	**%**	**No.**	**%**	**No.**	**%**
**Total number of deaths**	33	7.5	46	10.6	79	9.0
**Cause of deaths**						
**Prostate cancer**	2	0.5	2	0.5	4	0.5
**Primary cause of death (Not related to prostate cancer)**						
**Other primary malignancy**	10	2.3	18	3.9	28	3.2
**CVS - stroke**	3	0.7	2	0.5	5	0.6
**CVS - MI**	2	0.5	3	0.7	5	0.6
**CVS - PE**	1	0.2	4	0.9	5	0.6
**CVS Other**	3	0.5	1	0.2	4	0.5
**Bronchopneumonia**	2	0.5	1	0.2	3	0.3
**Other respiratory**	5	1.1	5	1.2	10	1.1
**Septicaemia**	1	0.2	2	0.5	3	0.3
**Other***	4	1.1	8	2.1	12	1.4

*CRT:2-unobtainable, 1-cerebral bleeding post coronography,1-old age
SBRT: 2-unobtainable, 5-unknown, 1-dementia

S9. Clinician reported toxicity at 5 years post treatment by treatment received

Worst RTOG toxicity at 5 years post treatment by treatment received

Worst Grade	GI		GU	
	CRT	SBRT	CRT	SBRT
	n (%)	n (%)	n (%)	n (%)
0	309 (87.0)	319 (90.1)	282 (79.4)	261 (73.5)
1	45 (12.7)	32 (9.0)	57 (16.1)	68 (19.2)
2	1 (0.3)	3 (0.8)	16 (4.5)	21 (5.9)
3	0 (0.0)	0 (0.0)	0 (0.0)	5 (1.4)
4	0 (0.0)	0 (0.0)	0 (0.0)	0 (0.0)
5	0 (0.0)	0 (0.0)	0 (0.0)	0 (0.0)
Missing	1	1	1	0
Grade 2+				
Yes	1 (0.3)	3 (0.8)	16 (4.5)	26 (7.3)
No	354 (99.7)	351 (99.2)	339 (95.5)	329 (92.7)
Chi-square (p)	0.31		0.11	
Fisher's Exact (p)	0.37		0.15	
Difference in proportion	-0.56%		-2.82%	
(95% CI)	(-1.67,0.54) %		(-6.28, 0.65) %	
p-value	0.31		0.11	

Worst CTCAE toxicity at 5 years post treatment by treatment received

Worst Grade	GI		GU		Erectile dysfunction	
	CRT	SBRT	CRT	SBRT	CRT	SBRT
	n (%)	n (%)	n (%)	n (%)	n (%)	n (%)
0	273 (76.5)	270 (76.1)	199 (55.7)	164 (46.2)	113 (38.2)	109 (36.8)
1	78 (21.8)	76 (21.4)	134 (37.5)	160 (45.1)	97 (32.8)	109 (36.8)
2	6 (1.7)	9 (2.5)	24 (6.7)	25 (7.0)	68 (23.0)	65 (22.0)
3	0 (0.0)	0 (0.0)	0 (0.0)	6 (1.7)	18 (6.1)	13 (4.4)
4	0 (0.0)	0 (0.0)	0 (0.0)	0 (0.0)	0 (0.0)	0 (0.0)
5	0 (0.0)	0 (0.0)	0 (0.0)	0 (0.0)	0 (0.0)	0 (0.0)
Missing	0	0	0	0	61	59
Grade 2+						
Yes	6 (1.7)	9 (2.5)	24 (6.7)	31 (8.7)	86 (29.1)	78 (26.4)
No	351 (98.3)	346 (97.5)	333 (93.3)	324 (91.3)	210 (70.9)	218 (73.6)
Chi-square (p)	0.43		0.32		0.46	
Fisher's Exact (p)	0.45		0.33		0.52	
Difference in proportion	-0.85%		-2.01%		2.70%	
(95% CI)	(-2.96, 1.26) %		(-5.9, 1.91) %		(-4.50,9.91) %	
p-value	0.43		0.32		0.46	

S10. Cumulative incidence of Grade 2+ events by 5 years

Grade 2+ events	Cumulative events n (%)	Estimated proportion by 5 years%	HR (95% CI)	p-values
**RTOG GU**				
CRT	76/433 (17.6)	18.3 (14.8, 22.5)	1.59 (1.18, 2.12)	<0.001
SBRT	109/415 (26.3)	26.9 (22.8, 31.5)		
**RTOG GI**				
CRT	45/433 (10.4)	10.2 (7.7, 13.5)	1.03 (0.68, 1.56)	0.94
SBRT	45/415 (10.8)	10.7 (8.1, 14.2)		
**CTCAE GU**				
CRT	126/433 (29.1)	29.8 (25.5, 34.5)	1.58 (1.25, 1.98)	<0.001
SBRT	175/415 (42.2)	42.5 (37.8, 47.5)		
**CTCAE GI**				
CRT	83/433 (19.2)	18.7 (15.2, 22.8)	0.96 (0.71, 1.31)	0.81
SBRT	77/415 (18.6)	18.8 (15.3, 23.0)		

Time to first occurrence of genitourinary and gastrointestinal grade≥2 toxicity event occurring up to 5 year after radiotherapy, assessed using RTOG and CTCAE

(A) RTOG Genitourinary

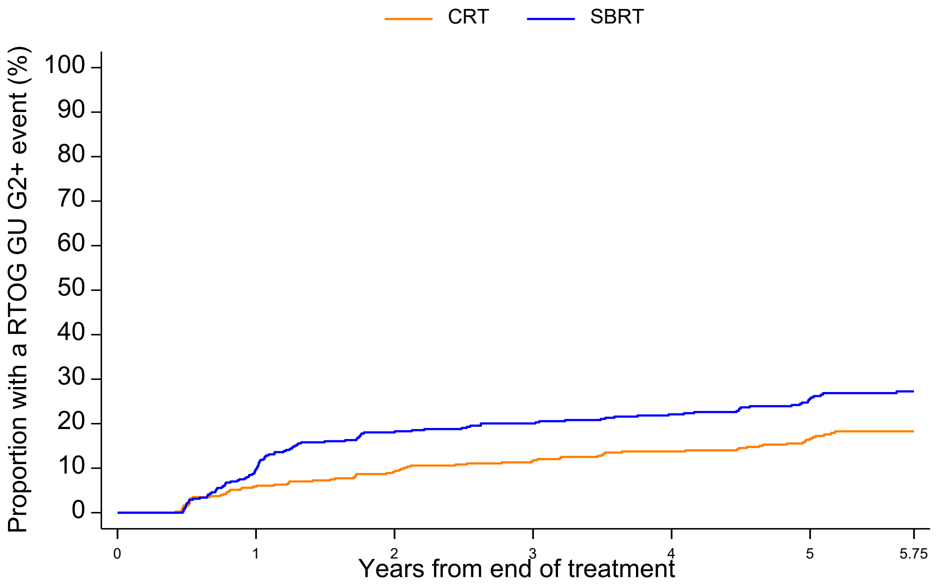

Number at risk (events)

	0		1		2		3		4		5		5.75
CRT	433	(25)	401	(14)	380	(10)	365	(9)	344	(11)	293	(5)	208
SBRT	415	(40)	371	(34)	329	(8)	316	(8)	302	(13)	254	(5)	185

(B) CTCAE Genitourinary

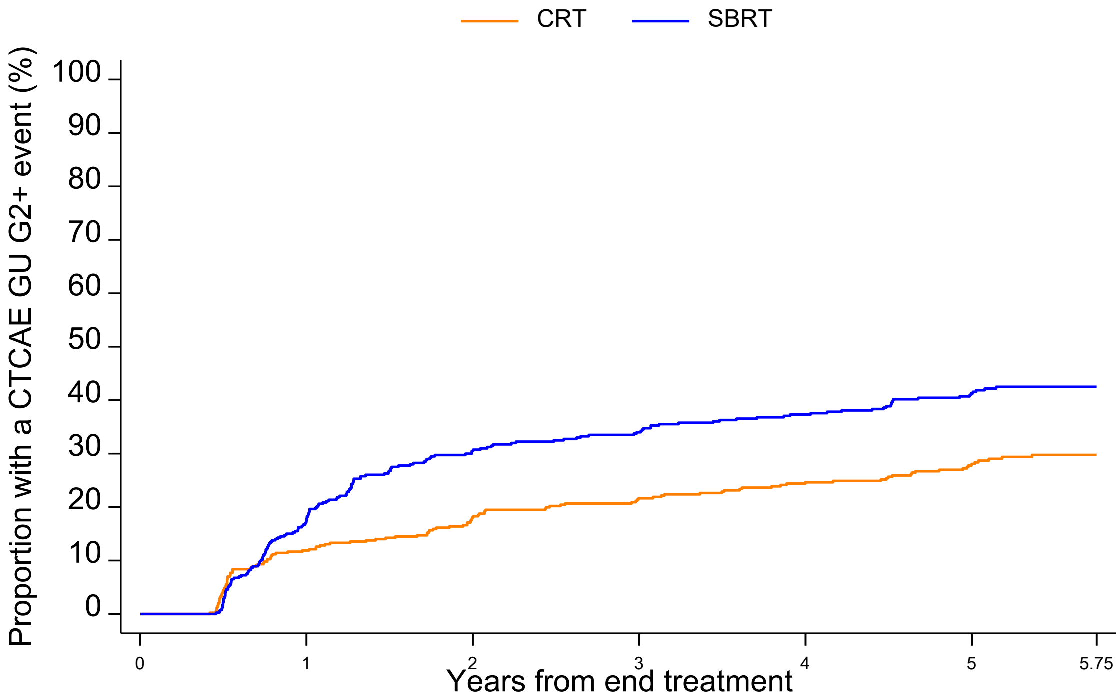

Number at risk (events)

CRT	433	(51)	375	(26)	344	(15)	322	(12)	299	(13)	252	(5)	178
SBRT	415	(73)	338	(52)	278	(14)	261	(13)	243	(15)	206	(4)	158

(C) RTOG Gastrointestinal

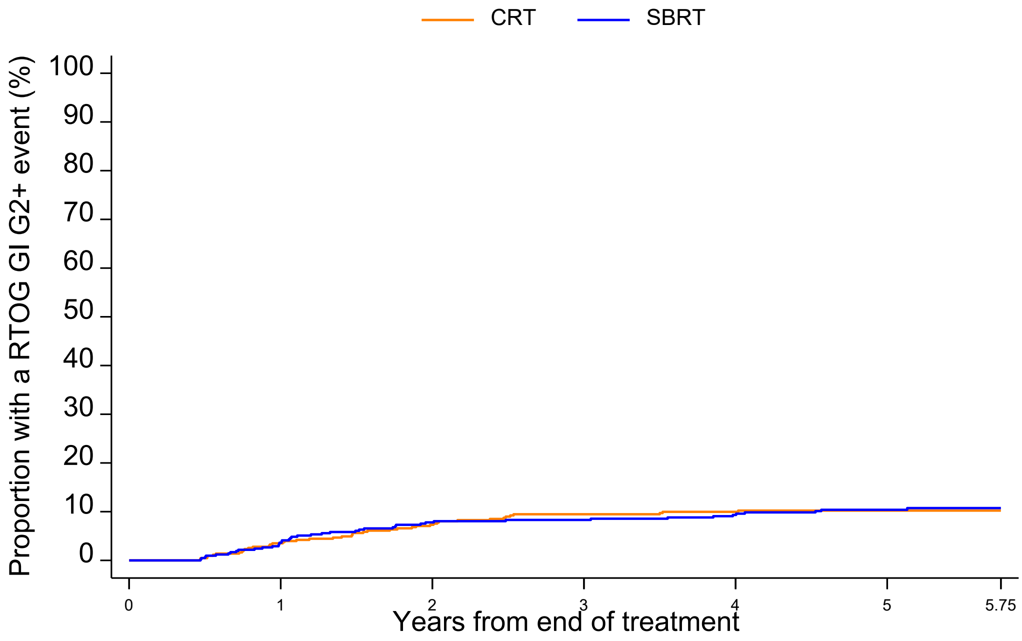

Number at risk (events)

CRT	433	(15)	411	(16)	386	(9)	373	(2)	358	(1)	310	(0)	220
SBRT	415	(15)	397	(17)	371	(2)	361	(4)	350	(4)	306	(1)	221

(D) CTCAE Gastrointestinal

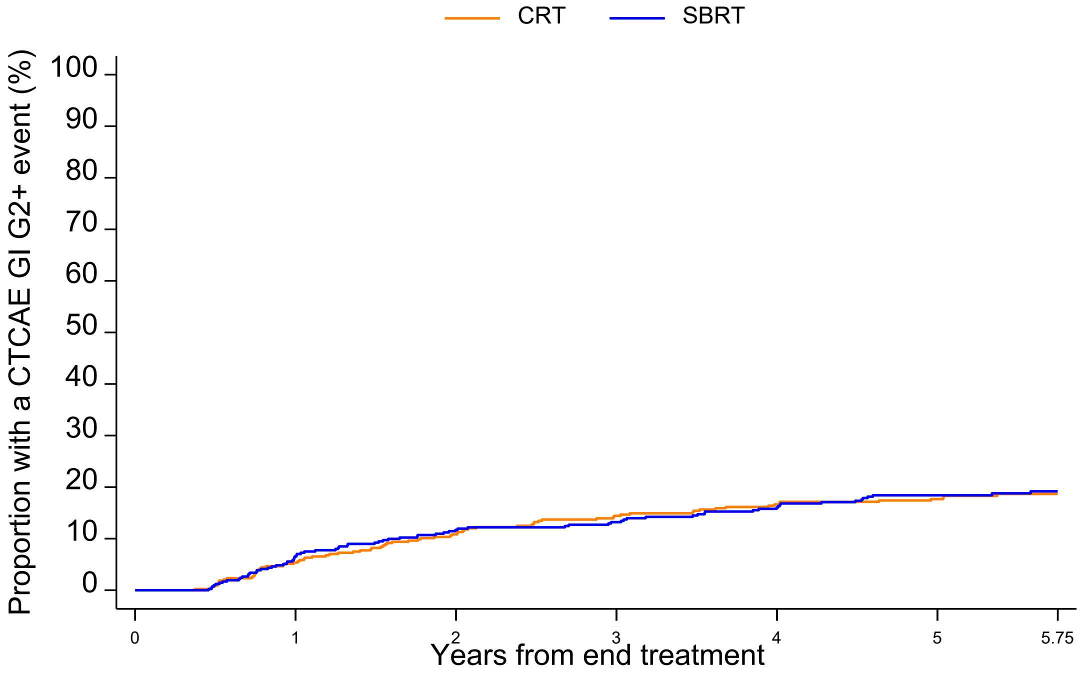

Number at risk (events)

CRT	433	(23)	403	(23)	373	(15)	353	(9)	333	(4)	288	(3)	203
SBRT	415	(27)	385	(21)	355	(6)	342	(11)	323	(9)	279	(2)	205

S11. Stacked bar charts illustrating distribution of genitourinary and gastrointestinal toxicity assessed using RTOG and CTCAE

(A) RTOG Genitourinary

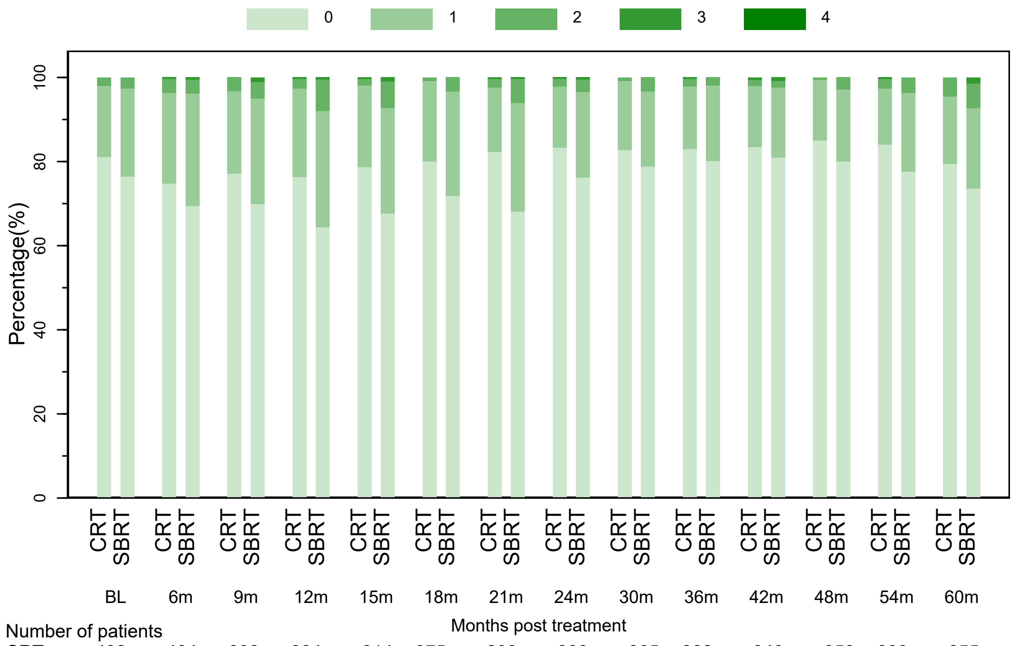

Number of patients

	BL	6m	9m	12m	15m	18m	21m	24m	30m	36m	42m	48m	54m	60m
CRT	402	404	398	384	314	375	299	383	365	382	340	353	339	355
SBRT	391	373	379	391	306	359	279	383	359	367	346	350	330	355

(B) CTCAE Genitourinary

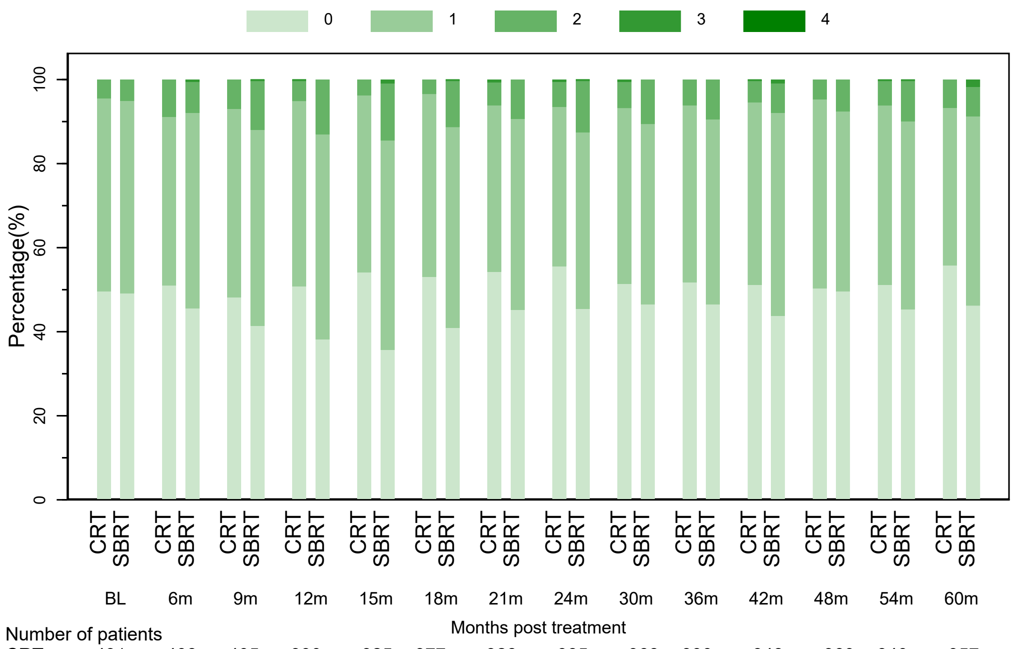

Number of patients	BL	6m	9m	12m	15m	18m	21m	24m	30m	36m	42m	48m	54m	60m
CRT	431	408	405	390	325	377	328	385	368	390	348	360	340	357
SBRT	413	380	384	393	317	362	321	383	359	372	352	355	333	355

(C) RTOG Gastrointestinal

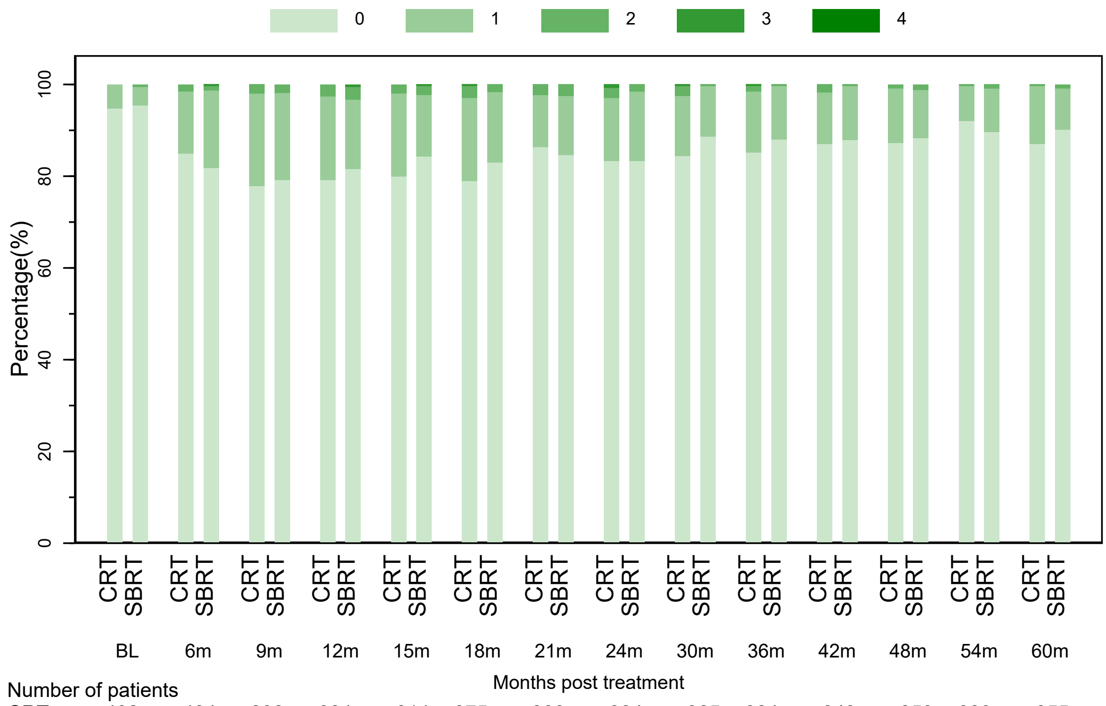

Number of patients	BL	6m	9m	12m	15m	18m	21m	24m	30m	36m	42m	48m	54m	60m
CRT	402	404	398	384	314	375	300	384	365	384	340	353	339	355
SBRT	391	374	379	391	306	359	279	383	360	368	347	350	330	354

(D) CTCAE Gastrointestinal

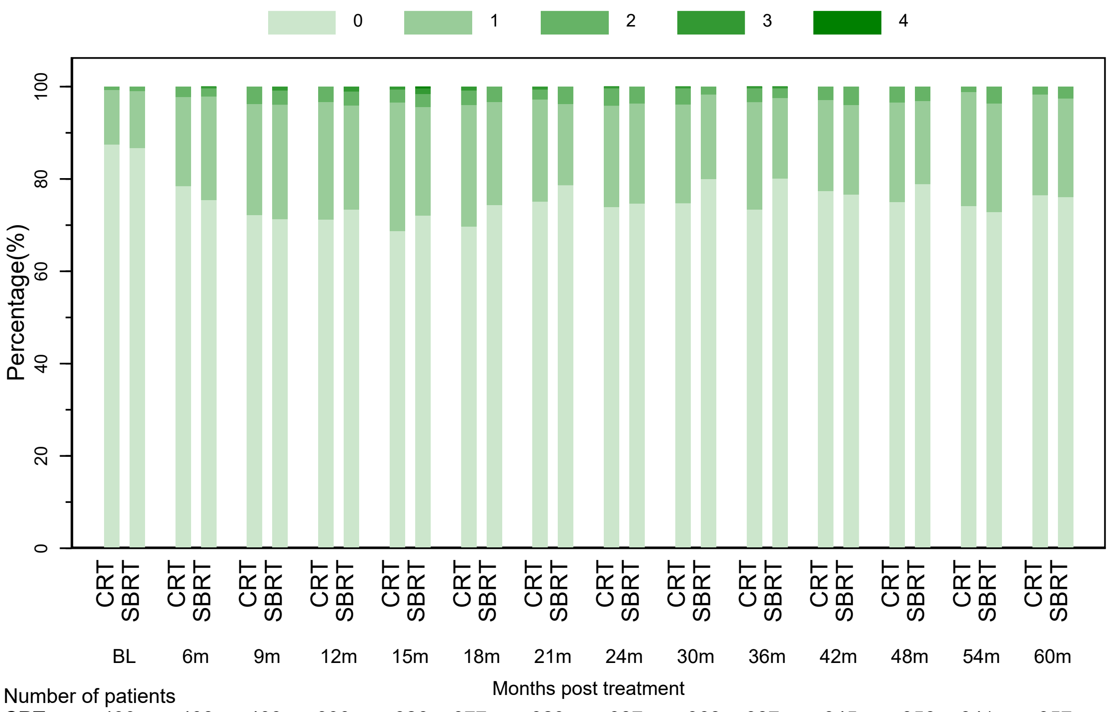

Number of patients	BL	6m	9m	12m	15m	18m	21m	24m	30m	36m	42m	48m	54m	60m
CRT	430	408	403	390	326	377	329	387	368	387	345	356	341	357
SBRT	413	379	383	394	316	363	323	384	360	372	351	355	335	355

S12. EPIC subdomain scores at baseline and 5 years by treatment received

EPIC-26 domain	Treatment received								Mann-Whitney
	CRT				SBRT				
	n	Median	IQR	Mean (SD)	n	Median	IQR	Mean (SD)	p-value
**Urinary incontinence**									
Baseline	398	100	(85.5, 100)	91.4 (14.7)	371	100	(85.5, 100)	91.3 (14.3)	0.75
5 years	241	100	(79.3, 100)	86.8 (18.3)	246	96.9	(73, 100)	84.4 (21.7)	0.45
**Urinary irritative/obstructive**									
Baseline	387	87.5	(81.3, 100)	87.7 (13.1)	360	87.5	(81.3, 100)	86.5 (13.9(	0.28
5 years	233	93.8	(81.3, 100)	88.1 (13.6)	233	93.8	(81.3, 100)	87.5 (14.1)	0.60
**Bowel Bother**									
Baseline	397	100	(95.8, 100)	95.9 (8.6)	375	100	(91.7, 100)	94.9 (8.6)	0.17
5 years	236	95.8	(87.5, 100)	90.4 (13.9)	237	100	(87.5, 100)	92.7 (10.9)	0.10
**Sexual Bother**									
Baseline	378	54.2	(27.8, 75)	53.0 (28.7)	363	48.9	(22.2, 77.8)	50.5 (29.6)	0.22
5 years	237	22.2	(16.7, 52.8)	34.0 (27.2)	235	26.3	(16.7, 57)	34.3 (27.2)	0.87
**Hormonal Bother**									
Baseline	398	100	(90, 100)	92.6 (10.9)	373	95	(90, 100)	92.2 (11.2)	0.62
5 years	229	95	(85, 100)	90.9 (13.3)	233	95	(85, 100)	89.7 (14.1)	0.35

EPIC subdomain scores range from 0 to 100 with higher scores indicating better quality of life.

Stacked bar charts illustrating the distribution of score for the EPIC-26 single item questions (A) 'Over-all how big a problem has your urinary function been?', (B) 'Overall, how big a problem has your bowel habits been' and (C) 'Overall, how big a problem was sexual function or lack of sexual function been'

(A) Overall urinary bother score

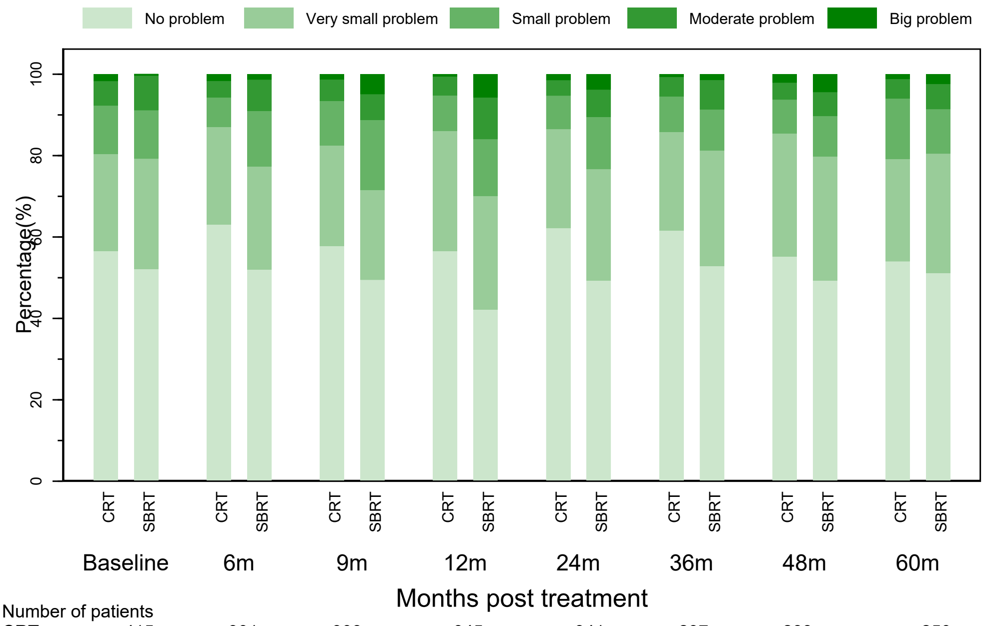

Number of patients								
CRT	415	301	308	345	341	297	288	250
SBRT	395	300	285	351	343	278	272	256

(B) Overall bowel bother score

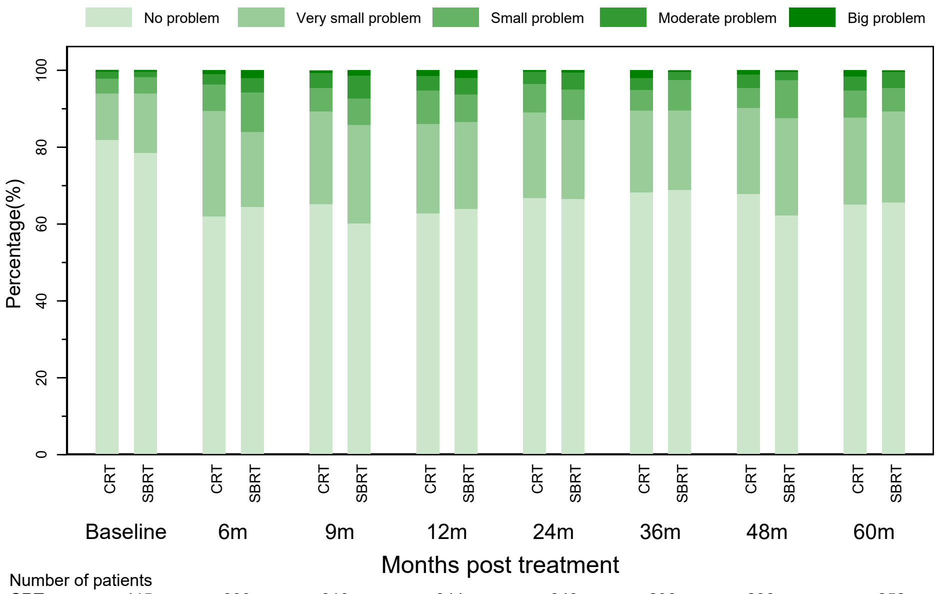

Number of patients								
CRT	415	303	310	344	340	296	286	252
SBRT	397	299	289	350	341	280	273	262

(C) Overall sexual bother score

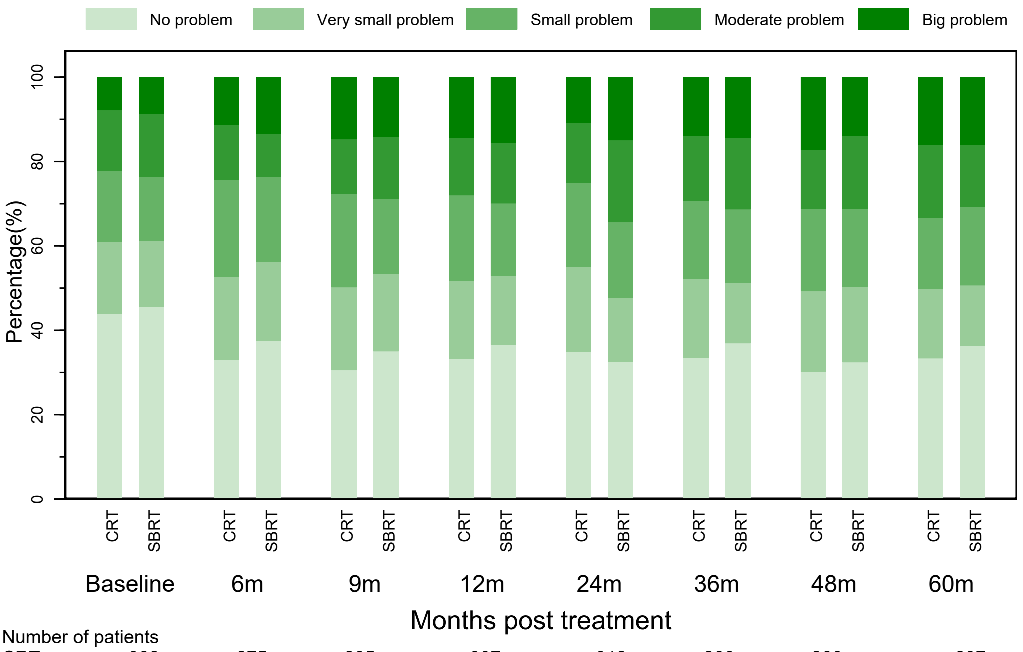

Number of patients	Baseline	6m	9m	12m	24m	36m	48m	60m
CRT	382	275	285	307	312	266	266	237
SBRT	367	270	260	314	314	252	250	237

S13. Summary of PACE Protocol Substantial Amendments

Amendment Number	Amendment Date	Summary	Response	Date of Response
1	03/12/2012	Changes to the medical physics paragraph to allow other centres to use cone beam CT to check that the prostate is in the correct position prior to treatment.	Approval	04/05/2023
2	08/08/2012	Increased patient number to 1716 patients to increase statistical power. Minor amendments to technical aspects of radiotherapy planning. Clarification of time windows for eligibility criteria and definition of biochemical failure.	Approval	09/18/2012
3	10/18/2012	Introduction of a patient information DVD that outlined the trial process for patients.	Approval	12/10/2012
4	01/15/2014	Sponsorship of the trial transferred from Accuray Incorporated (industry) to The Royal Marsden NHS Foundation Trust (academia).	Approval	02/07/2014
5	N/A	There is no amendment 5, it was incorrectly numbered with the label of amendment 6 when submitting the substantial amendment form.	N/A	N/A
6	08/06/2014	Relevant new sponsor changes added to the protocol.<br><br>Stereotactic body radiotherapy (SBRT) to be delivered on other (usually gantrybased) systems, rather than CyberKnife systems only.<br>Allowing fiducial markers of any type.<br><br>To permit image guidance by conebeam CT.<br><br>Heterogeneous 38Gy/4# SBRT treatment removed, as it was not expected that centres would use this technique.<br>TURP (transurethral resection of prostate) patients eligible.<br>MRI not mandated for planning or staging.<br>Exclusion criteria related to previous malignancy adjusted to reflect prolonged survival following many malignancies.<br>PSA failure definition adjusted to account for the "PSA bounce" phenomena.	Approval	10/24/2014

Amendment Number	Amendment Date	Summary	Response	Date of Response
		Follow up for efficacy endpoints measured from randomisation, rather than end of treatment.<br>Tissue donation, data sharing, and collection of NHS numbers (standard for UK trial patients) added. The Trial Executive Group abolished, as the sponsor was changed to an academic institution.		
7	06/22/2015	Change of database from Clinovo to Royal Marsden data collection systems.<br>Randomisation completed by the ICR-CTSU.<br><br>Addition of non-UK sites.<br><br>SAE reporting procedures updated in line with ICR-CTSU standard procedures.<br><br>Quality of Life booklets amended, the EPIC questionnaire replaced the PR25 as it was deemed more appropriate.<br>The following eligibility criteria was amended:<br>Biopsy requirements for patients on active surveillance described.<br>Bone scan requirements removed.<br>Prostate volume requirements measured within 6 months of randomisation removed.<br>Testosterone measurements beyond baseline removed.	**Approval**	**07/24/2015**
8	03/01/2016	Common and rare side effects of conventional radiotherapy or SBRT have been updated.<br>Changes made throughout the protocol, so sites were able to deliver either 78 Gy in 39 fractions OR 62 Gy in 20 fractions.	Approval	03/24/2016
9	11/16/2016	Allowed non-laparoscopic surgical methods in PACE-A.	Approval	12/13/2016

Amendment Number	Amendment Date	Summary	Response	Date of Response
10	07/18/2017	Change of primary endpoint for PACE-A to co-primary quality of life focused endpoints to consider side effects relevant to both surgery and SBRT incorporating urinary incontinence and bowel bother at the two-year timepoint. The original primary endpoint of biochemical/clinical recurrence became a secondary endpoint for PACE-A.<br><br>Revision of sample size based on new primary endpoints, from 858 (429 per treatment arm) to 234 (117 per treatment arm).	Approval	08/29/2017
11	08/22/2019	Addition of new PACE-C cohort, mirroring PACE-B but with the addition of 6 months androgen deprivation (as per standard of care) for men with high-intermediate and high risk localised disease.	Approval	10/25/2019
12	04/28/2020	In response to the COVID-19 pandemic, the protocol was amended to accommodate extended ADT treatment beyond 6 months (to maximum 18 months). The time frames with respect to ADT and staging assessments (MRI and pre-ADT PSA) were removed to avoid unnecessarily restrictive timelines not reflecting standard of care.<br><br>Granular details of the radiotherapy planning and delivery guidelines were moved from the protocol to a separate document- PACE Radiotherapy Planning and Delivery Guidelines.	Approval	06/25/2020
13	01/18/2022	Allowed participants to take part in the SPRUCE study within a trial for Quality of Life completion.	Approval	02/03/2022
14	11/06/2023	Changes to the wording in the protocol to add a new exploratory endpoint regarding translational studies and to clarify the opportunities for translational research in PACE.	Approval	11/15/2023

S14. Radiotherapy Planning and Delivery Guidelines

The following section describes the radiotherapy planning and delivery guidelines which has taken from the section 11 (Treatment) of the protocol

11.2 Evaluated Structures

11.2.1 The Clinical Target Volume (CTV):

For the purposes of this study, the CTV shall be defined as follows:

All patients:<br><br><u>Low risk</u>: CTV = prostate only (as defined on MRI planning scan where available)<br><br><u>Intermediate risk</u>: CTV = prostate plus proximal 1 cm of seminal vesicles from insertion point in the superior-inferior plane. This should include the middle ½ to ⅔ of seminal vesicle width (i.e. not the tips). Please contact the QA team for example contours.

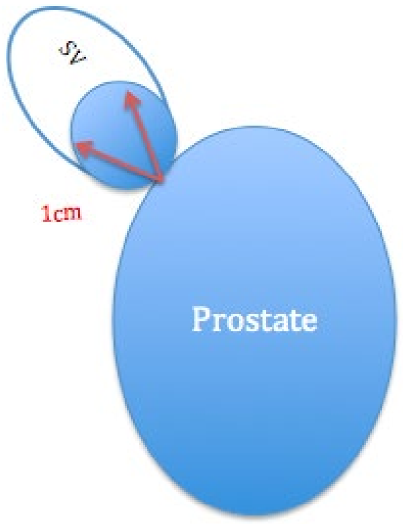

Figure 1: Schematic illustration of the seminal vesicle inclusion for PACE ("CTV" shown in blue)

11.2.2 The Planning Treatment Volume (PTV):

The CTV to PTV margins are different for prostate SBRT and conventional radiotherapy.

11.2.2.1 For conventional radiotherapy, margins will depend on the department's treatment delivery accuracy.

PTV margin for conventional radiotherapy:<br><br>PTV= CTV+ 5-9 mm, except 3-7 mm posteriorly

11.2.2.2 For prostate SBRT the PTV is defined as the CTV plus 4-5 mm, except posteriorly where the prostate abuts the rectum, where a 3-5 mm margin will be applied.

PTV margins for SBRT (36.25 Gy in 5 fractions)<br><br>PTV= CTV+ 4-5mm/ 3-5mm posteriorly

11.2.2.3 Planning volumes will be outlined and reported in line with ICRU 83 “Prescribing, recording and reporting photon-beam intensity modulated radiotherapy (IMRT)” where relevant.

11.2.3 Organs at Risk (OAR)

The following OAR will be contoured: these are given in reducing order of priority for planning constraints.

11.2.3.1 Rectum: defined as a solid structure, including the lumen and rectal wall, extending from the anus to the rectosigmoid junction.

11.2.3.2 Bladder: defined as a solid structure including the bladder wall and lumen.

11.2.3.3 Urethra if visible (prostate SBRT only): the prostatic urethra is defined as the lumen-mucosal interface, extending from bladder neck to the membranous urethra.

11.2.3.4 Penile bulb: the portion of the bulbous spongiosum that lies inferior to the urogenital diaphragm.

11.2.3.5 Femoral heads: Femoral heads are to be outlined from their most cranial aspect to the bottom of the curvature of the femoral head (i.e. exclude the femoral neck)

11.2.3.6 Bowel: Above rectum, within 15 cm of PTV for Cyberknife SBRT and within 4 cm PTV for gantry-based SBRT and IMRT. Bowel may be outlined as a ‘bowel bag’.

11.2.3.7 Testes: For CyberKnife SBRT, beams should not be allowed to traverse the testes, due to the effects on hormone production and subsequent confusion of biochemical outcomes. The bilateral testes should therefore be used as a ‘blocking structure’.

11.2.4 Structured naming convention for volumes

As an NCRN radiotherapy trial, the PACE study uses a standardised naming convention. This will avoid ambiguity and facilitate analysis of radiotherapy plan data. This convention is detailed in table 3.5.

Table 3.5: Structure naming convention for PACE

Volume	Naming convention
Conventional treatment volumes	
Clinical target volume: prostate +/- seminal vesicles	CTVp or CTVpsv

Planning target volume (receives 78 Gy or 62 Gy)	PTV_7800 or PTV_6200
SBRT treatment volumes	
Clinical target volume: prostate +/- seminal vesicles (receives 40 Gy)	CTVp_4000 or CTVpsv_4000
Planning target volume (receives 36.25Gy)	PTV_3625
Organs at risk	
Rectum	Rectum
Bladder	Bladder
Urethra	Urethra
Left femoral head	FemoralHead_L
Right femoral head	FemoralHead_R
Penile bulb	PenileBulb
Bowel	Bowel

11.3 Dose Specifications: (all specified doses are given over the entire course of treatment).

11.3.1 Conventional radiotherapy Dose Specifications:

11.3.1.1 Dose for the conventional arm will be either 78 Gy in 39 fractions daily over 8 weeks OR 62 Gy in 20 fractions daily over at least 27 days, and delivered using IMRT. The prescription dose shall be the dose to the PTV and the following dose objectives will be met: for 78 Gy: D98%≥74.1 Gy, D50%=78 Gy ± 1%, D2%≤83.5 Gy (aim for D2%<81.9 Gy): for 62 Gy: D98%≥58.9 Gy, D50%=62 Gy ± 1%, D2%≤66.3 Gy (aim for D2%<65.1 Gy). The minimum dose constraint (D98%) may be relaxed where necessary in order to meet the rectum high dose constraint, with limited undercoverage permitted posteriorly where PTV overlaps rectum.

11.3.1.2 Dose specifications for OAR are shown in Table 4.

Table 4: Dose Specifications for Conventional radiotherapy arm

Organ at risk	Dose volume constraints			
	Dose (Gy) for 78Gy/39 fractions	Dose (Gy) for 62Gy/20 fractions	Maximum Volume (% or cc)	
			Mandatory	Optimal
Rectum	30	24	-	80%
	40	32	-	65%
	50	40	60%	50%
	60	48	50%	35%
	65	52	30%	-
	70	56	25%	15%
	75	60	5%*	3%
Bladder	50	40	50%	-
	60	48	25%	-

	74	59	15%	5%
Femoral Heads	50	40	50%	5%
Bowel	50	40	17cc	-
Penile bulb	50	40	-	50%
	60	48	-	10%

* May require a reduction in posterior PTV margin and/or removal of rectal overlap from PTV during plan optimisation (as for SBRT planning).

11.3.2 Dose specifications for hypofractionated radiotherapy delivered with SBRT:

11.3.2.1 The dose for the SBRT arm will be 36.25 Gy given in 5 fractions over 1-2 weeks (i.e. daily or alternate daily).The prescription dose of 36.25 Gy shall be the dose to the PTV. V36.25 Gy to the PTV shall be greater than or equal to 95%. A secondary dose of 40 Gy should be delivered to the CTV (i.e. the prostate/SVs) such that the CTV V40 Gy is greater than or equal to 95%. For CyberKnife planning, the prescription isodose shall be 65-85% of Dmax (or 75-85% if urethra not contoured). For gantry-based SBRT, the following dose objectives should be met with respect to the PTV: D98%≥34.4 Gy, Dmax<48 Gy, and aim for D2%≤42.8 Gy, where possible. (A planning guide for gantry-based SBRT is available).

11.3.2.2 Dose specifications for OAR for SBRT are shown in Table 5. Minor and major variations are shown below.

Table 5: Dose Specifications for SBRT (36.25 Gy in 5 fractions)

OAR	Dose constraint
Rectum	V18.1 Gy <50% (i.e. 50% rectum <18.1 Gy)<br>V29 Gy <20 % (i.e. less than 20% rectum receiving 29 Gy)<br>V36 Gy <1cc
Bladder	V18.1 Gy <40%<br>V37 Gy <10cc (optimal V37 Gy <5cc)
Prostatic urethra (if visualised)	V42 Gy <50% (optimal, not mandatory)
Femoral head	V14.5 Gy <5%
Penile Bulb	V29.5 Gy <50%
Testicular	Blocking structure
Bowel	V18.1 Gy <5cc<br>V30 Gy <1cc

11.3.2.3 Rectum dose variations:

11.3.2.3.1 Minor variation: V36 Gy ≥ 1 cc, but < 2 cc.

11.3.2.3.2 Major variation:V36 Gy ≥ 2 cc

11.3.2.4 Bladder dose variations:

11.3.2.4.1 Minor variation: V37 Gy ≥ 10 cc, but < 20 cc.

11.3.2.4.2 Major variation: V37 Gy ≥ 20 cc

11.3.2.5 Target volume variations:

11.3.2.5.1 Minor variation: CTV V40 Gy 90-94.9%

11.3.2.5.2 Minor variation PTV: V36.25 Gy 90-94.9%

11.3.2.5.3 Major variation CTV: V40 Gy < 90%

11.3.2.5.4 Major variation PTV: V36.25 Gy < 90%

11.3.2.6 Investigators shall attempt to keep normal tissue doses and prescription coverage as close to "per protocol" specifications as possible. If all the above "per protocol" dose-volume criteria cannot be met on a given patient, then normal tissue constraints and target prescriptions may be relaxed to the "minor variation" range as follows: one minor variation in EITHER the primary or secondary dose prescription coverage (e.g. PTV V36.25 Gy 90-95% or CTV V40 Gy 90-95%) is allowed; two minor variations or one major variation is allowed only with the consent of the site chair.

11.3.2.7 Additional minor variation is allowed for constraints on the rectum and bladder. Major variations on OAR constraints are only allowed with the permission of the site chair. All variations shall be noted.

11.4 Radiotherapy plan data collection

11.4.1 Radiotherapy plan data will be collected (in DICOM format by electronic transfer) for all patients having radiotherapy within the trial. This data will be stored on a secure server by the sponsor.

11.5 Radiotherapy Treatment Delivery and Tracking

11.5.1 All radiotherapy techniques are to be approved in advance by the Chief Investigator and trials QA team.

11.5.2 It is highly recommended that radiotherapy start within 8 weeks of randomisation, but it must start within 12 weeks. Treatment will be given in a single phase over no more than 14 days for SBRT, no more than 61 days for conventional radiotherapy (78 Gy in 39 fractions), and 31 days for moderate hypofractionation (62 Gy in 20 fractions); longer planned treatment durations are to be discussed with the Chief Investigator for approval. In addition, for the 20 fraction treatment schedule overall time of treatment should be at least 27 days (as per CHHiP trial) and, in practice, means that these patients should start treatment on a Wednesday to Friday. Overall treatment duration will be recorded.

11.5.3 All patients will have image-guided radiotherapy, and it is strongly recommended that this is done with fiducial guidance. It is recommended that all patients be set up to fiducial markers prior to treatment and if a significant shift is required (>3 mm) the patient should be re-imaged after that shift. In addition, tomographic imaging pre-treatment is encouraged to rule out any significant changes in rectal position or prostate deformation.

11.5.4 At least three fiducials should be identified for each treatment. If fewer than three fiducials can be tracked, then additional fiducials can be placed, and the patient replanned. Where the ability exists rotational corrections should be made.

11.5.5 For SBRT using CyberKnife, patients will have fiducial-based intra-fraction motion corrected during treatment.

11.5.6 For SBRT with gantry based systems, it is anticipated that the majority of centres will use an arc-based IMRT technique, with or without flattening filter-free delivery. Flattening filter-free delivery should have a beam on time of under 3 minutes, in which case intra-fraction motion control is not mandated. Where beam-on time significantly exceeds 3 minutes, re-imaging should occur between beams/arcs (or at approximately 3-4 minute intervals). It is recommended that the couch is shifted for all displacement but it is mandatory to shift for any displacement ≥ 3 mm.

11.5.7 For centres using Calypso beacons or Elekta clarity ultrasound monitoring, prostate motion will be monitored continually and treatment paused (and position corrected) if prostate displacement exceeds 3 mm.

11.5.8 For gantry-based SBRT using tomographic imaging (i.e. cone beam CT) without fiducials, centres must demonstrate that they can deliver treatment to the required accuracy (given the significant prostate motion which may occur during treatment). This will be discussed and agreed on an individual centre basis with the Chief Investigator and trial QA team.

14 Quality assurance (QA)

14.2 Radiotherapy QA

The following QA documents and exercises must be completed by new centres for each radiotherapy treatment arm before commencing recruitment:

- Statement of unit calibration protocol
- Independent beam output audit
- Process document
- Benchmark case (see 14.2.1 below)
- IGRT benchmark test (conventional linac delivery only)
- Prospective individual case reviews will be performed for the first patient randomised to each treatment arm (see 14.2.2 below)

14.2.1 Benchmark Study: All potential sites shall receive, prior to patient enrolment, anonymous electronic patient data sets including CT and MRI images. A treatment plan shall be developed according to the protocol for both SBRT and IMRT, and the plan reviewed by the study team; completion of satisfactory benchmark plans is required prior to patient enrolment.

14.2.2 The first patient for each treatment allocation will undergo pre-treatment review. The treatment plan of the first patient enrolled at each site for each treatment must be reviewed prior to beginning treatment. The study team shall be notified at the time of enrolment of each patient, and of the proposed first treatment date, to assure the team's availability for review. There is the option for contours to be reviewed prior to planning if the centre prefers. After planning is complete, the treating site will make the treatment plan available to the study team site for review. The study team shall complete review within 2 weeks of receipt; treatment will only begin after any necessary corrections are

implemented and final plan is approved. In addition, the first intermediate risk case must also be reviewed if the cases reviewed above were both low risk and did not include the seminal vesicles.

14.2.3 Thereafter plans will be reviewed as deemed necessary by the study team.

14.2.4 All outlining should be either performed by or reviewed and approved by the PI at the centre who has been through the pre-trial outlining QA. Since this is a clinical trial and the patient numbers may not be excessive we hope this approach will be acceptable. However, where this is not feasible we recommend the following:

14.2.4.1 The PI should review and approve clinical outlines for the 1st 3 PACE patients recruited by each additional clinician at that centre, after which (assuming these are satisfactory) they are also approved for PACE. Note: Please ensure at least one is an intermediate risk group case, since many inconsistencies with proximal seminal vesicle outlining have been reported.

14.2.5 Should the PI leave and be replaced, the replacement should perform the PACE benchmark outlining QA to be reviewed by the PACE QA team.

14.2.6 Treatment plan exports

All patient treatment plans will be exported in DICOM format, anonymised, and sent to the RT QA team electronica
